# Perirenal adipose afferent nerves sustain pathological high blood pressure in rats

**DOI:** 10.1038/s41467-022-30868-6

**Published:** 2022-06-06

**Authors:** Peng Li, Boxun Liu, Xiaoguang Wu, Yan Lu, Ming Qiu, Yihui Shen, Yunfan Tian, Chi Liu, Xiru Chen, Chuanxi Yang, Mengqing Deng, Yaqing Wang, Jia Gu, Zhongping Su, Xuguan Chen, Kun Zhao, Yanhui Sheng, Shijiang Zhang, Wei Sun, Xiangqing Kong

**Affiliations:** 1 Department of Cardiology, The First Affiliated Hospital of Nanjing Medical University, 210029 Nanjing, China; 2 Cardiovascular Device and Technique Engineering Laboratory of Jiangsu Province, 210029 Nanjing, China; 3 Department of Cardiothoracic Surgery, The First Affiliated Hospital of Nanjing Medical University, 210029 Nanjing, China; 4 State Key Laboratory of Reproductive Medicine, Nanjing Medical University, 211166 Nanjing, China

**Keywords:** Hypertension, Hypertension

## Abstract

Hypertension is a pathological condition of persistent high blood pressure (BP) of which the underlying neural mechanisms remain obscure. Here, we show that the afferent nerves in perirenal adipose tissue (PRAT) contribute to maintain pathological high BP, without affecting physiological BP. Bilateral PRAT ablation or denervation leads to a long-term reduction of high BP in spontaneous hypertensive rats (SHR), but has no effect on normal BP in control rats. Further, gain- and loss-of-function and neuron transcriptomics studies show that augmented activities and remodeling of L1-L2 dorsal root ganglia neurons are responsible for hypertension in SHR. Moreover, we went on to show that calcitonin gene-related peptide (CGRP) is a key endogenous suppressor of hypertension that is sequestered by pro-hypertensive PRAT in SHRs. Taken together, we identify PRAT afferent nerves as a pro-hypertensive node that sustains high BP via suppressing CGRP, thereby providing a therapeutic target to tackle primary hypertension.

## Abstract (web summary)

The sympathetic nervous system can contribute to the development of hypertension, but the neurogenic mechanisms involved are incompletely understood. Here the authors report that afferent nerves in the perirenal adipose tissue (PRAT) contribute to the maintenance of high blood pressure, and PRAT ablation, denervation or upregulation of calcitonin gene-related peptide reduce blood pressure in hypertensive rats.

## Introduction

Hypertension is the most prevalent non-communicable disease, affecting 1.39 billion people worldwide^1^. Approximately 95% patients with high blood pressure have “essential” hypertension with undermined etiologies, and the high blood pressure (BP) can arise from combined actions of a variety of genetic, environmental, and behavior factors^2,3^. Disorders of neurogenic and body fluid regulation by the kidney are the most important underlying reasons of essential hypertension. Enhanced sympathetic nervous system activity and lower baroreflex sensitivity have been documented in the early stage and during the development of essential hypertension^4–6^. Moreover, studies of salt intake and hypertension have revealed the important role of the kidney in essential hypertension^7,8^. Although anti-hypertensive drugs targeting sympathetic system, fluid regulation, and vasodilatation have significantly reduced cardiovascular morbidity and mortality, no drug can cure essential hypertension or has a long-term anti-hypertensive effect by one-time treatment. On the other hand, life-long drug treatment has only achieved 28.4% BP control in high-income countries and 7.7% BP control in low- and middle-income countries, yet with significant side effects, polypharmacy, and noncompliance^1^. Pursuing alternative long-term therapeutic strategies is therefore urgently needed.

It is well-known that once chronic hypertension is installed at the early stage of essential hypertension, the high BP level is maintained, even in the absence of increased sympathetic nerve activity^9^. The kidney plays an important role in the maintenance of high BP in essential hypertension^10^. However, neurogenic mechanism involved in this regulation remains largely unknown. Perirenal adipose tissue (PRAT), also known as the adipose capsule of kidney, is a layer of fatty material surrounding the kidney and lies inside the renal fascia. PRAT is neutral fat consisting of white adipose tissue and islands of brown adipose tissue^11,12^. Similar to the nerve supply of pure white adipose tissue, efferent nerves of PRAT are only composed of sympathetic nerves of autonomic nervous system^13^. Adjacent to PRAT, pararenal fat is a collection of pure white adipose tissue, located superficially to the renal fascia. Both PRAT and pararenal adipose tissues (PaRAT) are part of retroperitoneal fat. Prospective cohort studies have shown that visceral especially retroperitoneal obesity is robustly associated with hypertension^14,15^. It has also been noted that renal adipose tissues, including PRAT and PaRAT, are associated with high BP in patients with obesity^16,17^.

In prior studies, sensory nerves were discovered to act as a connector between adipose tissue and high BP in rats fed with a high-fat diet^18^. Sensory nerve fibers within adipose tissue, marked by the differential expression of calcitonin gene-related peptide (CGRP), carry chemical sensory stimuli toward neural soma that lie in dorsal root ganglia (DRGs) and then central nervous system. Sympathetic nerve activities were subsequently activated in obese hypertensive rats. This neural reflex was termed as “adipose afferent reflex”^18^. However, roles of sensory nerve fibers of adipose tissue in other pathological high BP remain unclear.

Here, we investigate whether and how PRAT regulates hypertension in spontaneous hypertensive rats (SHRs), a wildly used animal model mimicking human essential hypertension. We show that PRAT ablation (PRATA) or denervation (PRATD) effectively and stably reduces high BP, without affecting normal BP. Further, we uncover, by gain- and loss-of-function studies, that L1-L2 DRG neurons and their sensory fibers projecting to PRAT play a critical role in maintaining high BP via neuron remodeling and sequestering CGRP.

## Results

### Effects of PRATA on BP and neuroendocrine activity

**Fig. 1:**
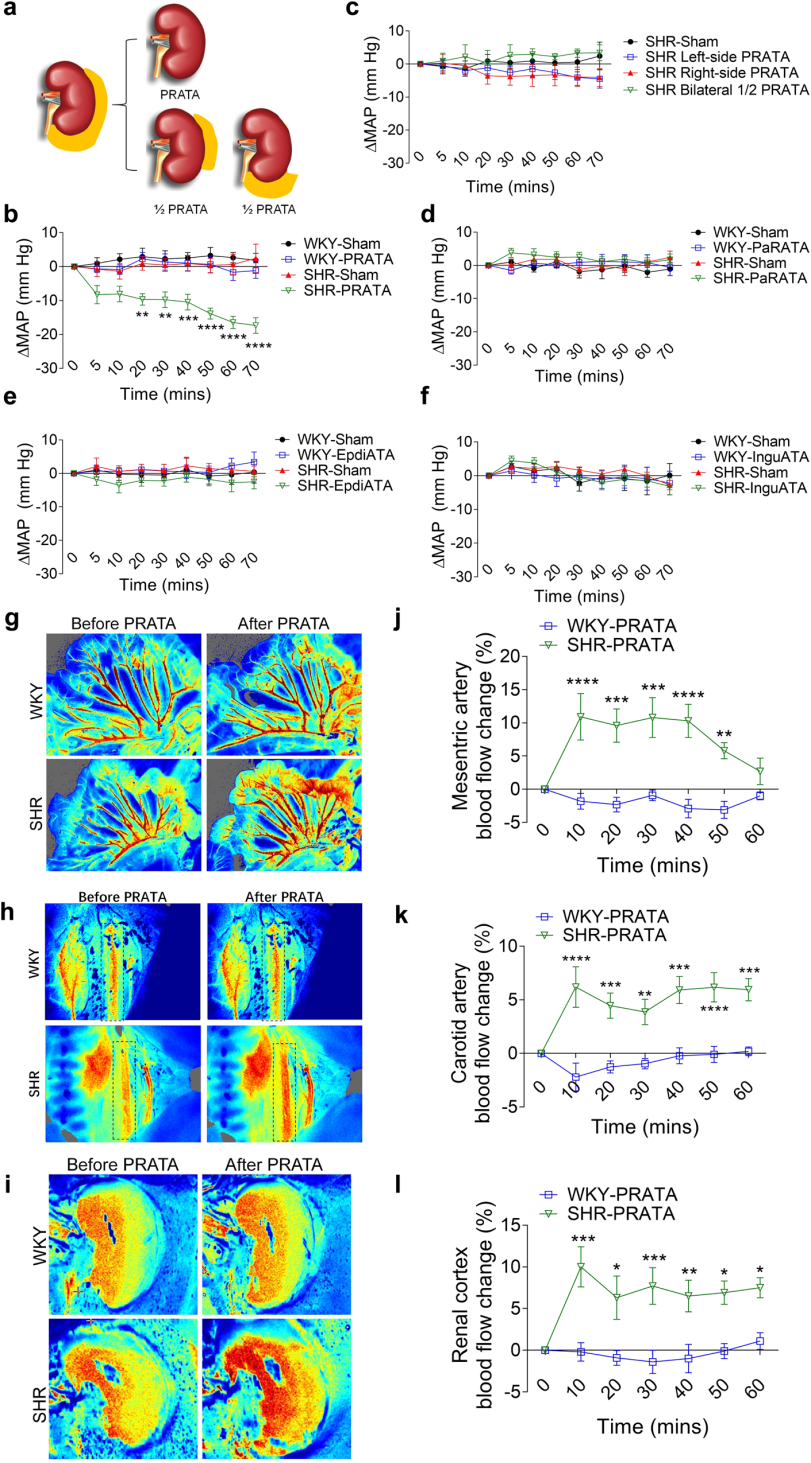
Acute effects of PRATA on blood pressure and peripheral vasomotion. **a** Surgical diagram of PRATA. **b** Acute changes of the intracarotid arterial MAP post PRATA, *n* = 6 per group; Data are mean ± SEM. **P < 0.01, ***P < 0.001, ****P < 0.0001. Analysis of variance (ANOVA), Bonferroni post-hoc test, two-sided. **c**–**f** Changes of intracarotid arterial MAP post unilateral and half-volume PRATA (**c**), pararenal adipose tissue ablation (**d**), epididymal adipose tissue ablation (**e**) and inguinal adipose tissue ablation (**f**), *n* = 6 per group; Data are mean ± SEM. *P* > 0.05. ANOVA, Bonferroni post-hoc test, two-sided. **g**–**l** Laser speckle contrast imaging of the blood flow of mesenteric artery (**g**), carotid artery (**h**) and renal cortex (**i**), and quantification (**j**–**l**). *n* = 5 per group for all the imaging of the blood flow experiments in (**j**) and (**l**). *n* = 7 for WKY-PRATA group, *n* = 5 for SHR-PRATA group in (**k**). Data are mean ± SEM. *P < 0.05, **P < 0.01, ***P < 0.001, ****P < 0.0001. ANOVA, Bonferroni post-hoc test, two-sided.

To examine the role of PRATA in BP control, we surgically ablated PRAT in SHRs and normotensive Wistar-Kyoto (WKY) rats, without affecting the renal sinus, ureter and adrenal glands (schematic diagram shown in Fig. 1a, photographs of anatomy and operation shown in Supplementary Fig. 1a). PRATA acutely decreased intracarotid mean arterial pressure (MAP) in SHRs, but not control WKY rats, within 20 min after surgical procedure (Fig. 1b). In contrast, removal of unilateral or half-volume PRAT, PaRAT, epididymal or inguinal adipose tissue in SHRs did not affect MAP (Fig. 1c to f), indicating a tissue-specific effect of PRATA in lowering the elevated BP in SHRs. Additionally, PRATA did not affect body weight and lifespan (Supplementary Fig. 2a, b), suggesting limited systemic side effects.

**Fig. 2:**
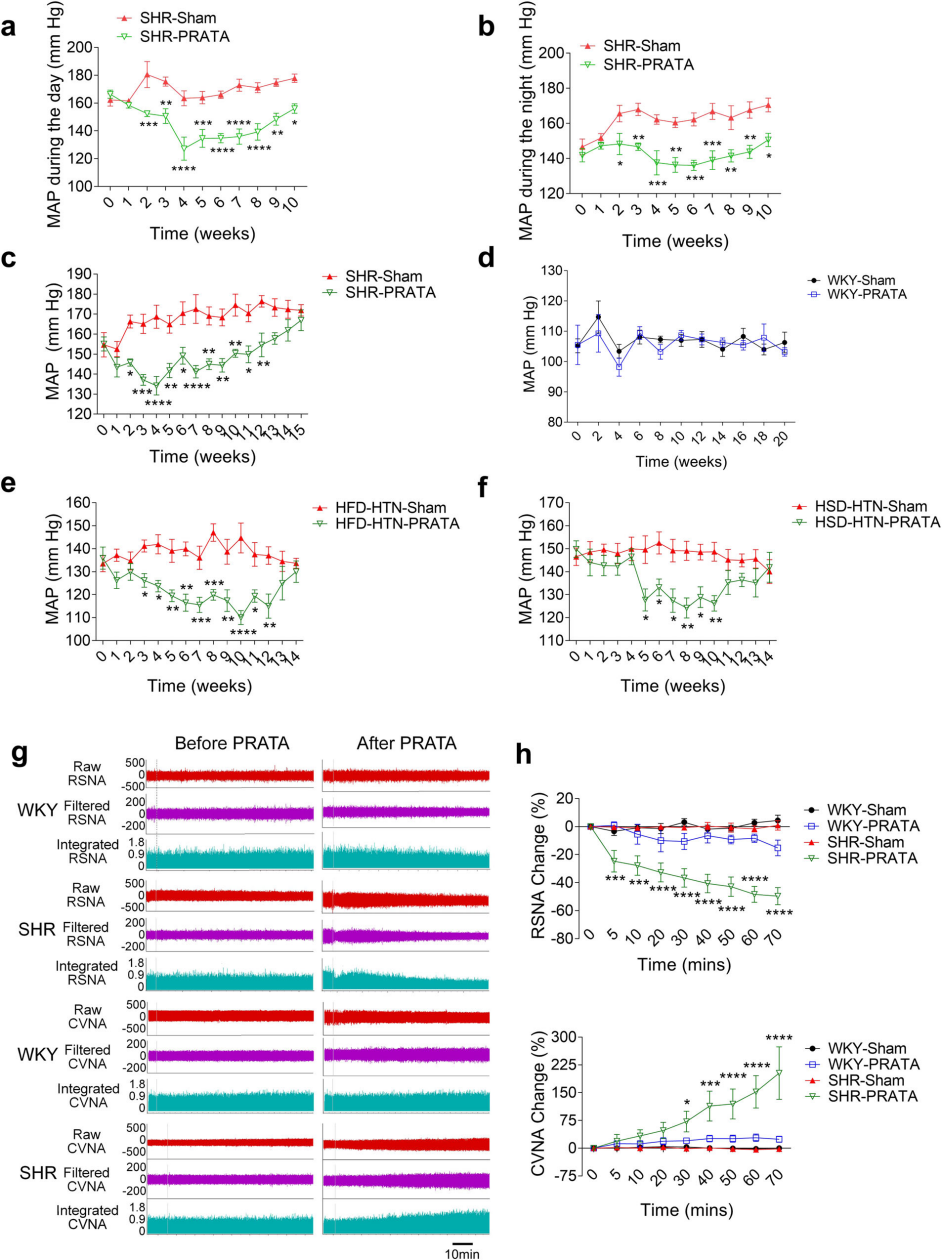
Effects of PRATA on hypertension of different causes and sympathetic output. **a**, **b** Weekly measurement of arterial mean BP post PRATA in SHRs using radiotelemetry in the daytime (**a**) and at night (**b**), *n* = 6 per group; Data are mean ± SEM. *P < 0.05, **P < 0.01, ***P < 0.001, ****P < 0.0001. ANOVA, Bonferroni post-hoc test, two-sided. **c**, **d** Weekly measurement of tail arterial BP post PRATA in SHR (**c**) and WKY rats (**d**), *n* = 7 per group; Data are mean ± SEM. *P < 0.05, **P < 0.01, ***P < 0.001, ****P < 0.0001. ANOVA, Bonferroni post-hoc test, two-sided. **e**, **f** weekly measurement of tail arterial BP post PRATA in high fat diet (HFD) induced hypertensive rats (**e**) and high salt diet (HSD) induced hypertensive rats (**f**), *n* = 7 per group; Data are mean ± SEM. *P < 0.05, **P < 0.01, ***P < 0.001, ****P < 0.0001. ANOVA, Bonferroni post-hoc test, two-sided. **g**, **h** Changes of renal sympathetic and cervical vagal nerve discharge (**g**) and quantification (**h**), *n* = 6 per group; Data are mean ± SEM. *P < 0.05, ***P < 0.001, ****P < 0.0001. ANOVA, Bonferroni post-hoc test, two-sided.

Next, we used the laser speckle contrast imaging to examine blood flow of mesenteric artery and renal cortex vessels after PRATA. We found that PRATA led to significant dilatation of mesenteric arteries (Fig. 1g) and carotid arteries (Fig. 1h), increased renal cortex blood flow (Fig. 1i), as well as undulant dilatation of the dorsalis pedis artery (Supplementary Fig. 2c), 10 min after the procedure. Weekly BP monitoring using implanted radiotelemetry revealed that PRATA-induced reduction of MAP of SHRs during the day and night (Fig. 2a, b). Similar results were obtained using tail-cuff measurement of BP that PRATA reduced the MAP of male (Fig. 2c) and female (Supplementary Fig. 2f) SHRs, but had no effect on BP in male (Fig. 2d) and female (Supplementary Fig. 2g) WKY rats. The BP level was similar between radiotelemetry and tail-cuff measurement (Supplementary Fig. 3a). Importantly, ablation of half-volume PRAT, PaRAT, epididymal or inguinal adipose tissue in SHRs did not show long-term effects on BP (Supplementary Fig. 3b–e).

To further address whether PRAT-induced BP-lowering effects is limited in SHRs, we performed PRATA in high salt and high fat diet-induced hypertension rat models. PRATA showed similar reduction of high BP in these models (Fig. 2e, f). Taken together, the acute and chronic BP-lowering effect is presented in different hypertensive rat models after PRATA. The presence and integrity of PRAT plays an important role in the maintenance of high BP, whereas the baseline volume or mass of PRAT is not associated with the increase of BP.

### PRATA reduces L1-L2 DRG neuron function and promotes neuron remodeling

To investigate the neuroendocrine mechanism(s) by which PRATA suppresses high BP, we focused on catecholamine metabolism. We found that PRATA did not affect serum levels of epinephrine, norepinephrine and dopamine (Supplementary Fig. 4a–c), urine catecholamines and related metabolites in both SHRs and WKY rats (Supplementary Fig. 4d–h) or renal cortex level of norepinephrine (Supplementary Fig. 4i). We next explored whether PRATA affected sympathetic or parasympathetic output by examining the changes of renal sympathetic nerve (RSN) and cervical vagal nerve (CVN). We found that RSN activity was rapidly and significantly decreased within 5 min after PRATA in SHRs, whereas only a slight, but not significant, decrease was observed in WKY rats. CVN activity was significantly elevated 30 min after PRATA in SHRs, but not WKY rats (Fig. 2g, h).

We then examined whether PRATA decreased BP by inhibiting angiotensin-II (Ang II) signaling. Our results showed that no significant change of Ang II level was observed in renal cortex or medulla after PRATA (Supplementary Fig. 4j, k). PRATA did not reduce the increased BP induced by Ang II (Supplementary Fig. 4l). Thus, the chronic BP-lowering effect of PRATA is unrelated to the reduction of Ang II signaling.

**Fig. 3:**
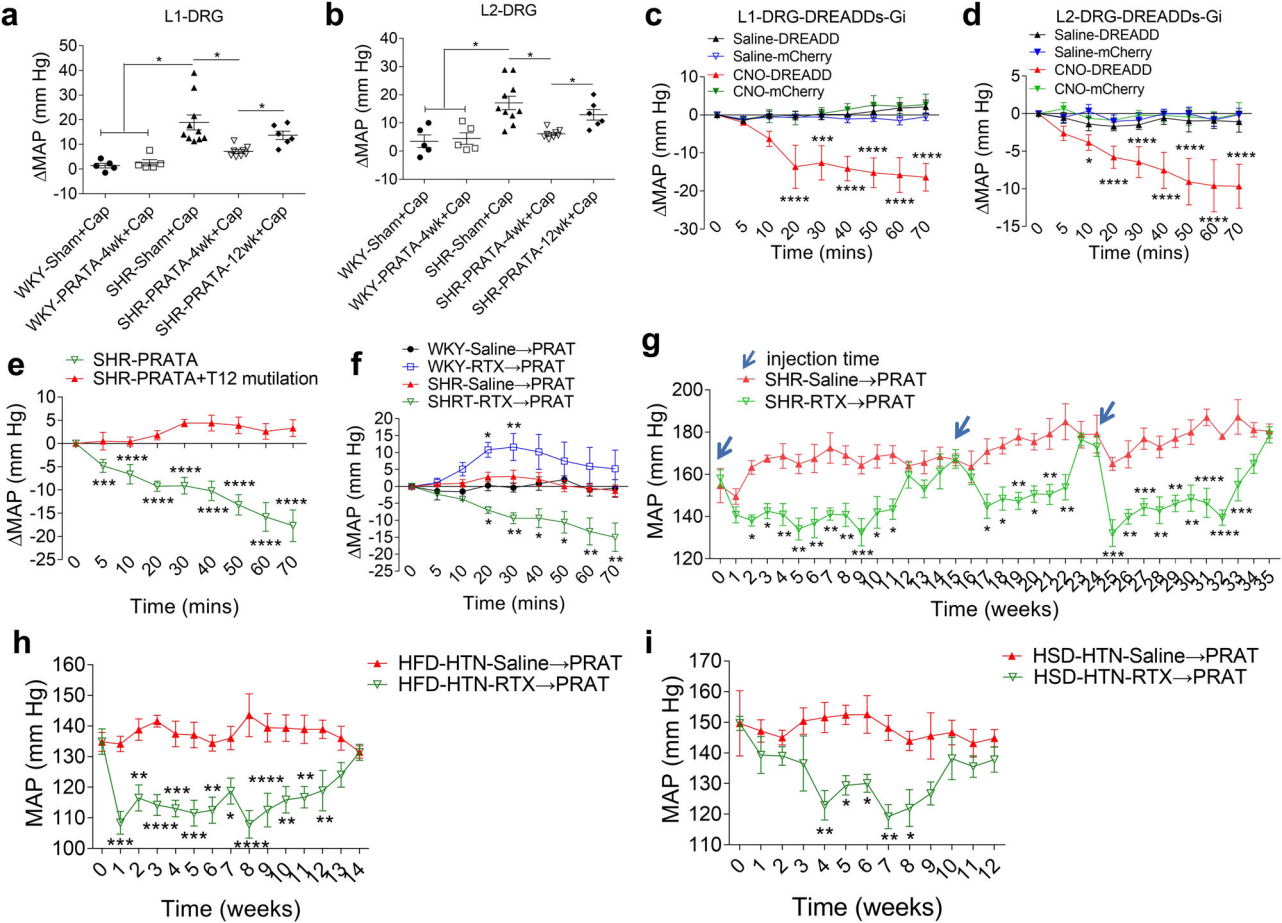
Enhanced L1-L2 DRG neuron activity as a regulator for high BP. **a**, **b** Changes of intracarotid arterial MAP under L1-L2 DRG injection with capsaicin post PRATA, *n* = 5 for WKY-Sham + Cap and WKY-PRATA-4wk + Cap groups, *n* = 10 for SHR-Sham + Cap group, *n* = 8 for SHR-PRATA-4wk + Cap group, *n* = 6 for SHR-PRATA-12wk + Cap group; Data are mean ± SEM. *P < 0.05. ANOVA, Bonferroni post-hoc test, two-sided. **c**, **d** Changes of the intracarotid arterial MAP after selective inhibition of neuronal activity of L1-L2 DRGs using Gi-DREADDs in SHR, *n* = 5 for Saline-DREADD and CNO-DREADD group, *n* = 6 for Saline-mCherry and CNO-mCherry group; Data are mean ± SEM. *P < 0.05, ***P < 0.001, ****P < 0.0001 compared with Saline-DREADD group. ANOVA, Bonferroni post-hoc test, two-sided. **e** Changes of the intracarotid arterial MAP post PRATA with or without T12 spinal cord mutilation, *n* = 6 for SHR-PRATA group, *n* = 5 for SHR-PRATA + T12 mutilation group; Data are mean ± SEM. ***P < 0.001, ****P < 0.0001. ANOVA, Bonferroni post-hoc test, two-sided. **f** Changes of the intracarotid arterial MAP post PRAT denervation by RTX in SHRs, *n* = 6 per group; Data are mean ± SEM. *P < 0.05, **P < 0.01. ANOVA, Bonferroni post-hoc test, two-sided. **g** Weekly measurement of tail arterial BP post three repeated PRATD by RTX in SHRs, *n* = 6 per group; Data are mean ± SEM. *P < 0.05, **P < 0.01, ***P < 0.001, ****P < 0.0001. ANOVA, Bonferroni post-hoc test, two-sided. **h**, **i** Weekly measurement of tail arterial BP post PRATD in HFD induced hypertensive rats (**h**) and HSD induced hypertensive rats (**i**), *n* = 8 for HFD-HTN-Saline→PRAT group, *n* = 7 for HFD-HTN-RTX → PRAT group in (**h**); *n* = 7 per group in (**i**); Data are mean ± SEM. *P < 0.05, **P < 0.01, ***P < 0.001, ****P < 0.0001. ANOVA, Bonferroni post-hoc test, two-sided. Saline→PRAT: injection of saline into perirenal adipose tissue, RTX → PRAT: injection of resiniferatoxin into perirenal adipose tissue.

Next, we went on to address potential effects of PRATA on neuronal regulation of blood vessels. PRAT is not a ganglion and cannot directly affect the sympathetic output and vascular tone. The activity of sympathetic neural circuit and vascular tone depend on the function of sensory nerves in peripheral organs^18^. On these grounds, we then focused on the affected neurons in DRG, the most peripheral sensory ganglia of PRAT, and their potential roles in high BP. Here we confirmed that T13-L2 are the predominant DRGs projecting sensory nerves to PRAT in SHRs (Supplementary Fig. 5a), which is similar to that in Sprague-Dawley (SD) rats in our previous studies^19^. Gain-of-function study by directly injecting a low dosage of capsaicin (a sensory nerve activator at a low dosage) into T13-L3 DRGs showed that T13-L2 but not L3 DRGs injection caused a strong increase of MAP (~18 mmHg) within 5 min in SHRs, compared to moderate elevation (~6 mmHg) in WKY rats (Fig. 3a, b; Supplementary Fig. 5b, c). Intra-DRG injection of capsaicin-induced MAP elevation was blunted in SHRs at 4 weeks after PRATA, and this inhibitory effect was sustained for 12 weeks (Fig. 3a, b). The expression of TRPV1, a receptor of capsaicin, was increased in the L1-L2 DRGs of SHRs, which was downregulated after PRATA (Supplementary Fig. 5d–g). Conversely, a loss-of-function chemogenetic study was performed using Designer Receptors Exclusively Activated by Designer Drugs (DREADDs^20^) as a selective inhibitor of neuronal activity of L1-L2 DRGs that innervate PRAT (Supplementary Fig. 6a, b). Ten minutes after injecting clozapine-N-oxide (CNO), the specific ligand for the inhibitory receptor M4Di, intracarotid arterial MAP began decreasing in SHRs. The amplitude for MAP decrease was up to 20 mmHg, while there is no significant change in MAP in control rats (Fig. 3c, d).

### Sensory nerves within PRAT maintain the high BP in SHRs

The results from acute, direct manipulation of DRG neurons suggest that PRATA improves the abnormally augmented DRG neuron function to sustain high BP. Blockade of BP-lowering effect of PRATA by a simultaneous T12 spinal cord mutilation further supports the essential role of sensory nerves in PRAT (Fig. 3e). To disrupt the connection between PRAT and DRG neurons, we next selectively damaged the sensory nerves in PRAT (PRATD) by injecting resiniferatoxin (RTX), a sensory nerve specific neurotoxin, into PRAT in SHRs and WKY rats (Supplementary Fig. 7a). RTX injection into PRAT led to an acute decrease in MAP in SHRs but not WKY rats (Fig. 3f), without affecting the afferent nerve activity of the kidneys (Supplementary Fig. 2e). This BP-lowering effect of PRATD lasted for more than 10 weeks in SHRs (Fig. 3g), but not in WKY rats (Supplementary Fig. 7b). Repeated injection of RTX in the same rats at 15 weeks and 24 weeks after the first dose of RTX decreased the recurrent high BP, and this effect lasted for additional 8–9 weeks (Fig. 3g). We also noted significant reduction of BP in high fat and high salt diet-induced hypertensive rats by PRATD (Fig. 3h, i), and a moderate reduction of BP in SHRs following RTX treatment of neighboring PaRAT that mainly received L2 DRG neuron projecting nerves (Supplementary Fig. 7c, d). Taken together, the presence of bilateral PRAT sensory nerves from the L1-L2 DRGs is required for the sustained BP elevation in SHRs.

### PRATA promotes changes of neuronal gene expression and remodeling

**Fig. 4:**
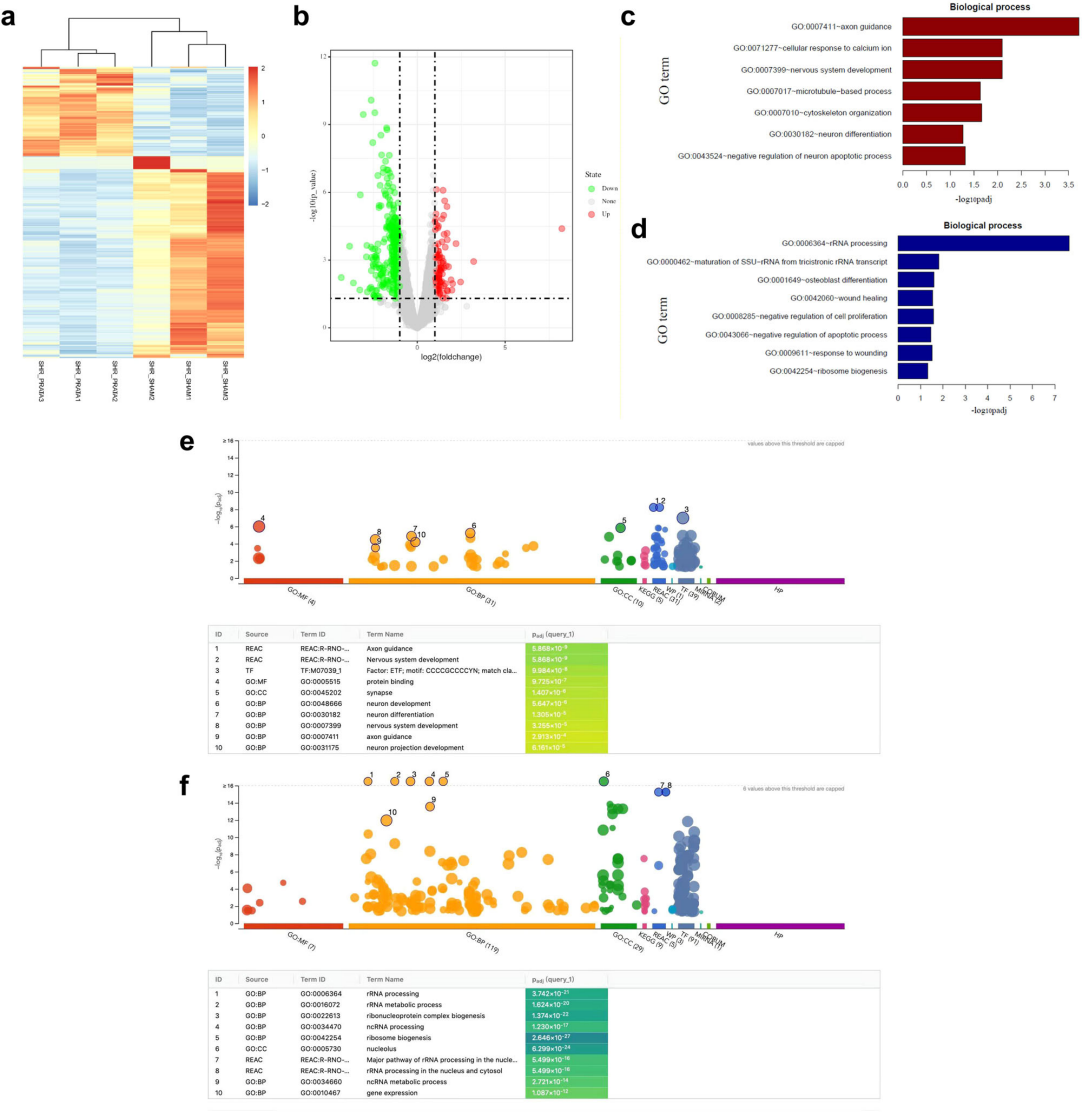
Whole-transcriptome sequencing of isolated L1-L2 DRG neurons in SHRs. **a**, **b** Differential gene expression in L1-L2 DRG neurons 4 weeks post PRATA. Hierarchical cluster and volcano analysis of 433 genes expressed differentially between sham and PRATA groups. The expression levels are indicated by the increased gradient of dark blue to light blue to red intensity. **c**, **d** GO enrichment analysis shows most of those upregulated genes are associated with neuron plasticity and development, and those downregulated genes are prone to RNA processing and would healing. **e**, **f** Test of differential expressed gene abundances using g:Profile showed that up-regulated genes mainly involved in neuron activities, while the downregulated genes enriched for RNA activities in post-PRATA SHRs compared with sham-operated SHRs.

To further explore the mechanism of PRATA regulating DRG neuron function and/or remodeling, we performed whole-transcriptomic analysis in isolated primary L1-L2 DRG neurons 4 weeks after PRATA. We found that 133 genes were upregulated and 300 genes were downregulated after PRATA (Fig. 4a, b). Most upregulated genes were related to neuronal development and plasticity (Fig. 4c), whereas most downregulated genes were related to wound healing, cell proliferation and apoptosis (Fig. 4d). Notably, the upregulated genes include *MAP2* (microtubule associated protein 2, a well-known molecular marker for dendrite formation) and *CALCA* (calcitonin gene-related peptide, also abbreviated as CGRP, a major neuromodulator in sensory nerves^21^). Moreover, functional enrichment of up/downregulated genes between sham-operated and post-PRATA SHRs further showed that up-regulated genes mainly involved in neuron activities, while the downregulated genes enriched for RNA activities (Fig. 4e, f).

**Fig. 5:**
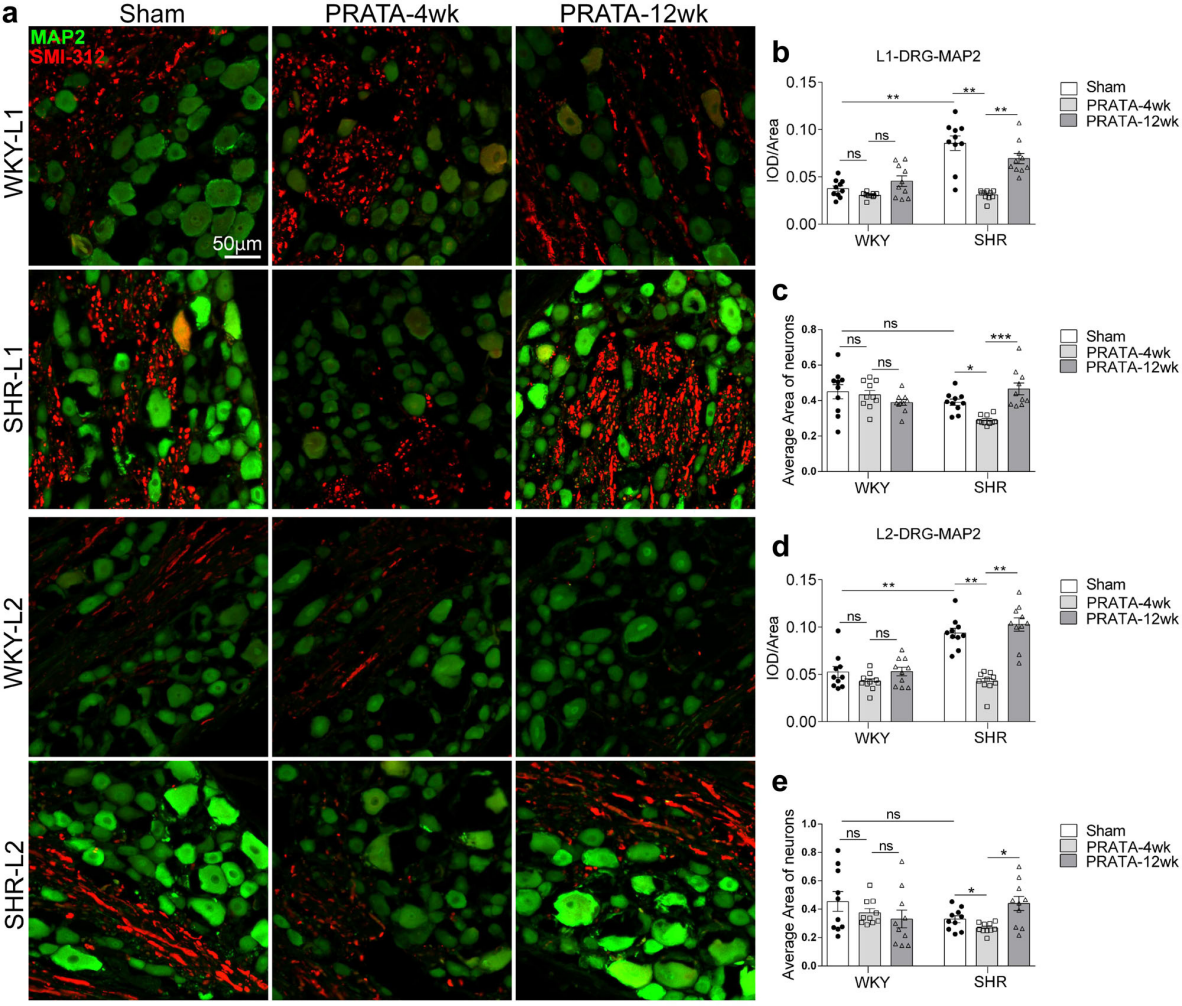
PRATA promotes L1-L2 DRG neuron remodeling. **a**–**e** Imaging of central and peripheral axons by immunostaining of MAP2 and SMI-312 in L1-L2 DRG (**a**), scale bar = 50 μm; and quantification (**b**–**e**). *n* = 10 per group, Data are mean ± SEM. Non-significant (ns) *P* > 0.05, *P < 0.05, **P < 0.01, ***P < 0.001. ANOVA, Bonferroni post-hoc test, two-sided.

We then analyzed the status of axons and dendritic shape at L1-L2 DRGs using immunostaining with MAP2 and SMI-312 (a pan-axonal neurofilament marker monoclonal antibody). In contrast to the increased MAP2 mRNA level, MAP2 protein and mean size of neurons from L1-L2 DRG neurons remained lower and smaller than those before PRATA in SHRs at 4 weeks after PRATA, but these changes were reversed at 12 weeks after PRATA (Fig. 5). We further analyzed the ultrastructure of L1 DRG neurons and found that the number and size of microtubules were decreased after PRATA in SHRs (Supplementary Fig. 8). Collectively, these data suggest that PRATA changes the dendritic structure and function of DRG neurons, whereas recovery of dendritic shape of DRG neurons possibly terminates the BP-lowering effects of PRATA.

### CGRP acts as a key factor mediating PRATA induced BP-lowering effects

**Fig. 6:**
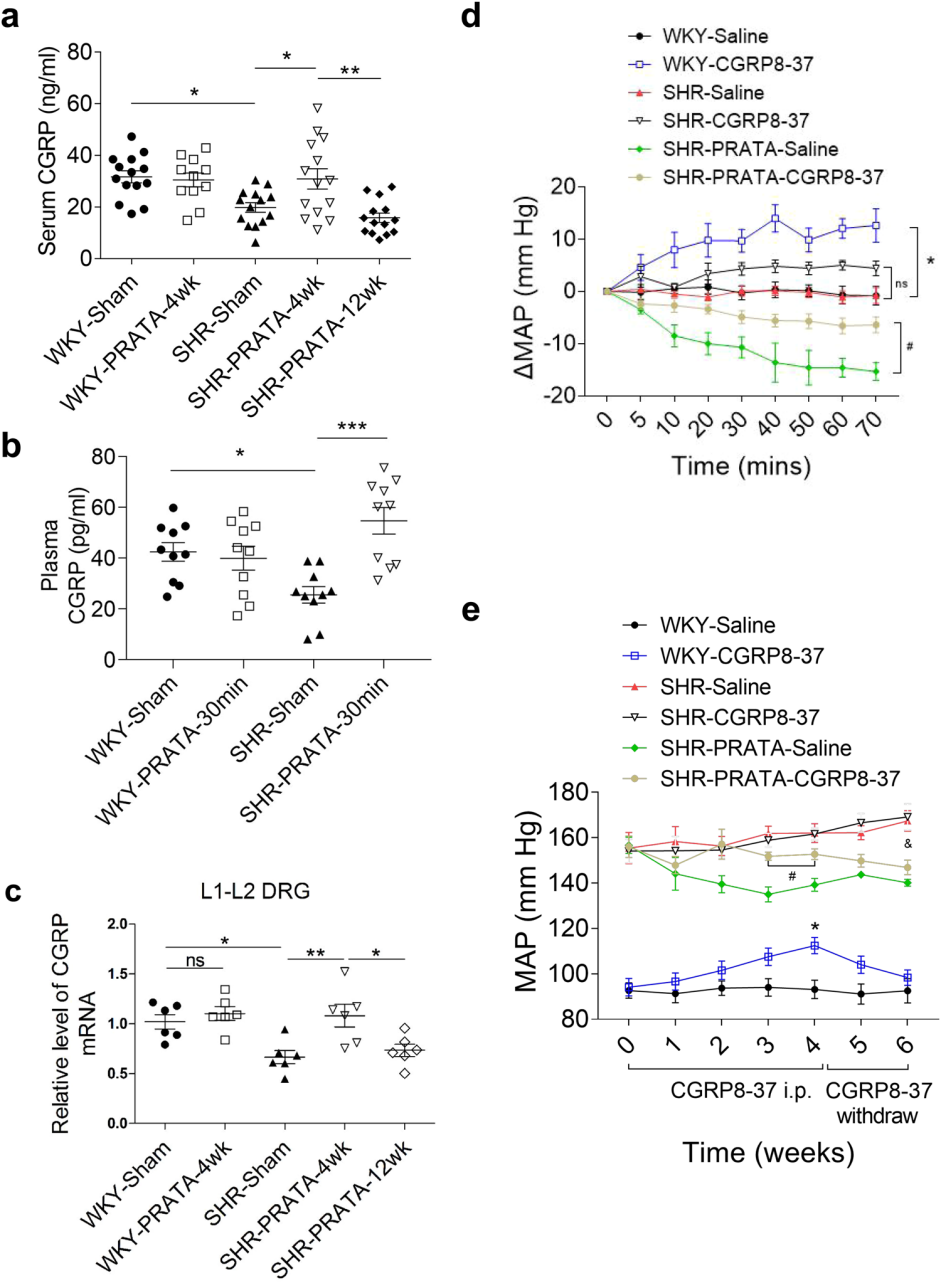
CGRP as a mediator for BP-lowering effects of PRATA. **a**, **b** Serum or plasma levels of CGRP assessed by ELISA post PRATA in chronic (**a**) or acute (**b**) experiments. *n* = 11 for WKY-PRATA-4wk group, *n* = 14 for other groups in (**a**). *n* = 10 per group in (**b**); Data are mean ± SEM. *P* > 0.05 non-significant (ns), *P < 0.05, **P < 0.01, ***P < 0.001. ANOVA, Bonferroni post-hoc test, two-sided. **c** mRNA levels of CGRP assessed by qPCR in L1 and L2 DRGs post PRATA, *n* = 6 per group; Data are mean ± SEM, *P* > 0.05 non-significant (ns), *P < 0.05, **P < 0.01. ANOVA, Bonferroni post-hoc test, two-sided. **d** Changes of the intracarotid arterial MAP post PRATA combined with CGRP8-37 (1 mg/kg) treatment, *n* = 5 for WKY-Saline, WKY-CGRP8-37, SHR-PRATA-Saline groups, *n* = 6 for SHR-Saline, SHR-CGRP8-37, SHR-PRATA-CGRP8-37 groups; Data are mean ± SEM. *P < 0.05, WKY-Saline vs. WKY-CGRP8-37; ^#^*P* < 0.05, SHR-PRATA-Saline vs. SHR-PRATA-CGRP8-37; *P* > 0.05 non-significant (ns), SHR-Saline vs. SHR-CGRP8-37. ANOVA, Bonferroni post-hoc test, two-sided. **e** Weekly measurement of tail arterial BP post PRATA combined with CGRP8-37 (10 μg/kg/d) treatment, n = 8 for WKY-CGRP8-37 group, n = 7 for other groups; Data are mean ± SEM. *P < 0.05, WKY-Saline vs. WKY-CGRP8-37; ^#^*P* < 0.05, SHR-PRATA-Saline vs. SHR-PRATA-CGRP8-37; ^&^*P* < 0.05, SHR-Saline vs. SHR-PRATA-CGRP8-37. ANOVA, Bonferroni post-hoc test, two-sided.

As mentioned above, CGRP-encoding gene *Calca* was upregulated in post-PRATA DRG neurons. Synthesized mainly by DRG neurons, CGRP acts as a potent vasodilator after being secreted by sensory nerve terminals^22–24^. To address whether CGRP acts as a key factor mediating the decrease of BP, we measured the levels of CGRP in serum, DRG, spinal cord and mesenteric artery after PRATA. Our results showed that SHRs had a reduced level of CGRP at spinal cord at L1-L2, but not L3 (Supplementary Fig. 9a–f). PRATA restored the CGRP level at 4 weeks after the procedure, which declined to basal level by 12 weeks after PRATA (Supplementary Fig. 9a–f). A parallel change was also noted in the serum and mesenteric arteries after PRATA (Fig. 6a, b; Supplementary Fig. 10). Notably, the number of CGRP positive neurons in L1-L2 DRGs was not affected by PRATA (Supplementary Fig. 11a–d), while there was an increase in CGRP mRNA expression (Fig. 6c) and the number of CGRP (+) ARC (+) neurons (Supplementary Fig. 11e–h) at 4 weeks after PRATA, which was consistent with the increased release of CGRP in L1-L2 DRGs. Furthermore, we showed that a CGRP antagonist, CGRP8-37, increased the MAP of WKY rats and partially reversed the BP-lowering effect of PRATA in SHRs, while withdrawal of CGRP8-37 restored the effect of PRATA (Fig. 6d, e). These results support that a decreased level of CGRP is required for sustaining high BP in SHRs.

## Discussion

It is known that enhanced sympathetic neural activation is more closely associated with the level of abdominal visceral fat than total fat mass or subcutaneous fat deposition in human individuals with obesity^25^. Indeed, direct activation of sensory nerves within white adipose tissue has identified that adipose sensory nerve mediated afferent reflex increases the sympathetic outflow and promotes short-term high BP in obese rats. However, whether visceral adipose tissue is involved in primary hypertension remains unknown. In this study, we provide direct evidence supporting PRAT as a pathological homeostatic control site for BP control, which sustains the high BP via a neuronal afferent circuit involving CGRP, albeit not affecting normal BP. Our data demonstrate that PRAT, not other fat pads such as inguinal, pararenal or epididymal adipose tissue, is associated with the maintenance of high BP in essential hypertension.

Neurogenic dysregulation, particularly elevated sympathetic output and altered baroreflex sensitivity, is part of mechanism underlying essential hypertension^4–6,26,27^. However, changes of these neuronal activity are also involved in physiological regulation of BP, which may lead to hypotension. The sensory nerves with peripheral tissues in BP regulation are not fully understood. Among the sensory nerves, TRPV1(+) neurons are of great interest. TPRV1 is traditionally recognized as a nociceptive ion channel that is activated by exogenous physical stimuli (temperature>43 degrees Celsius, acidic conditions, etc.) These neurons could also be activated by endogenous metabolites (linoleic acids metabolites, arachidonic acid metabolites, endocannabinoid, etc.) and agonists from natural sources (capsaicin, RTX, etc.) Systemic destruction of sensory nerves by high dose of capsaicin in newborn rats indicates that sensory nerves dysfunction impairs renal function and leads to salt-sensitive high BP^28,29^. In contrast to the non-specific denervation of sensory nerves, our results of PRAT denervation by repeated RTX treatments leading to sustained reduction of high BP support a specific role of PRAT sensory nerves in the maintenance of high BP in SHRs. Previous studies have demonstrated that sensory renal nerves do not contribute to the development and maintenance of hypertension in SHRs^30,31^. In contrast to the role of sensory renal nerves, we found that the main corresponding sensory neurons of PRAT on hypertension are located at L1-L2 DRGs which is different from those of kidney at T9-T13^32^. Our data suggest that the effect of PRAT sensory nerves on adult high BP is not through the kidney, even though PRAT surrounds the kidney. Whether PRAT is involved in the development of hypertension in SHRs needs further investigation.

We uncover functional and structural changes of DRG neurons which project sensory nerves to PRAT as the functional base of sensory nerve mediated high BP control. Gain-of-function study with capsaicin or loss-of-function study with DREADD and spinal cord mutilation allowed us to identify that L1-L2 DRG neurons independently regulate high BP. Similar BP-lowering effect of PRATA, PRATD or direct chemogenetic DRG manipulation indicates that nerve terminal disruption is sufficient to induce the loss-of-function of DRG neurons, leading to decreased high BP. However, whole-transcriptome sequencing and histology analysis of L1-L2 DRGs demonstrate a powerful self-recovery capacity of DRG neurons after PRATA/PRATD, characterized by the increased expression of neuron development and plasticity genes after PRATA. These data explain the non-permanent BP-lowering effect of PRATA, as PRATA knockdown of DRG neuron function is reversible. Nonetheless, our findings indicate that BP-lowering effect of PRATA has a desirable long-lasting consequence, though not permanent.

Although various previous studies showed that plasma concentration of CGRP could be reduced, unchanged or increased in patients with hypertension^33–35^, previous studies have reported that deletion of CGRP gene disables endogenous anti-hypertensive mechanism, thus considering CGRP as an effective BP-lowering agent^36–38^. Likewise, decreased neuronal and serum levels of CGRP was also discovered in SHRs^39^. Thus we proposed a causative or compensatory role of CGRP in hypertension. In this study, whole-transcriptome sequencing of L1-L2 DRG neurons shows that CGRP can directly regulate vascular dilatation and BP. Our study reveals that PRATA leads to long-term reduction of high BP, at least partly through increasing CGRP production by L1-L2 DRG neurons. We propose that two possible factors may contribute to the increase of circulating level of CGRP in acute and chronic BP-lowering effect. In acute effect, injury and partial degeneration of sensory nerve axons after PRATA may facilitate the leakage of CGRP. In chronic effect, PRATA/PRATD upregulates CGRP expression in L1-L2 DRG neurons, which results in the increased circulating level of CGRP. Despite this, remodeling of DRG neurons after surgical ablation reverses this beneficial remodeling process and decreases CGRP expression and release, resulting in restoration of high BP. In this way, our data support that (i) serum CGRP may be a potential biomarker for noninvasively monitoring the effect of PRATA; (ii) PRAT sensory neurons may be a potential therapeutic target for reducing high BP in clinic; and (iii) plasma CGRP may be a candidate marker to evaluate this potential therapeutic strategy.

Different from most previous studies that have focused on the sympathetic part of neural regulation of high BP control^5,6,40^, our work addresses the nociception sensory part including sensory nerves and DRG neurons, and demonstrates a previously unrecognized role of sensory nerves of DRG neurons in maintaining high BP. Importantly, we demonstrate that PRATA does not affect lifespan and shows no effect on normal BP in animals, which is consistent with the absence of major side effects after PRAT excision in normotensive patients who receive a radical nephrectomy^41,42^.

**Fig. 7:**
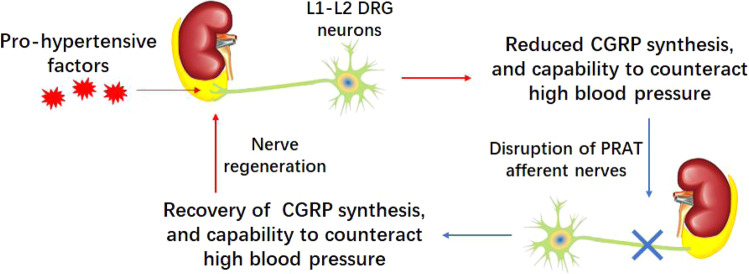
Schematic illustration of this study. PRAT afferent nerves serve as a pathological node of hypertension that sustains high BP via suppressing CGRP, thereby being a potential therapeutic target to tackle primary hypertension.

Thus, our study thereby provides a rationale for the use of PRATA or selective PRATD to control clinical essential hypertension, especially for patients who have reduced plasma CGRP or are resistant to conventional BP-lowing medicines (Fig. 7).

## Methods

### Animals and ethics statement

Spontaneously hypertensive rats (SHR), Wistar-Kyoto rats (WKY) (12 weeks old, weighing about 250 g) and Sprague-Dawley (SD) rats (6 or 9 weeks old, weighing about 170 g or 310 g) were purchased from Vital River Biological Co., Ltd (Beijing, China). All animal experiments were approved by the Experimental Animal Care and Use Committee of Nanjing Medical University and performed in accordance with the Guide for the Care and Use of Laboratory Animals (NIH publication No. 85–23, revised 1996). Animals were housed under regular 12 h dark: light cycles and given standard food and water.

High fat diets (HFD) experiments were performed as described previously^18^. Specifically, SD rats (9 weeks old, weighing 300–320 g) were randomly assigned to receive a control chow (Ctrl) diet (12% kcal as fat) or HFD (42% kcal as fat) for 12 weeks. Rats with greater weight gains were defined as obese rats. Rats with systolic blood pressure (SBP) ≥ 150 mm Hg were referred to as hypertension.

High salt diets (HSD) experiments were performed as described previously^43^. Specifically, SD rats (6 weeks old, weighing 160–180 g) were randomly assigned to receive a control chow (Ctrl) diet (0.4% salt) or HSD (8% salt) for 8 weeks. Rats with SBP ≥ 150 mm Hg were referred to as hypertension.

### Blood pressure measurement

For acute BP measurement, rat was anesthetized by isoflurane and consistently ventilated with the rodent ventilator (Harvard Apparatus, MA, USA). The right carotid artery was isolated, and a polyethylene tube (PE50) was cannulated as previously described^44–47^. A PowerLab data acquisition system (ABP, AD Instruments, Sydney, Australia) was applied for continuous recording of mean arterial blood pressure (MAP).

Implanted radiotelemetry was used in chronic BP measurement for <12 weeks. The rat was anesthetized by isoflurane and consistently ventilated with the rodent ventilator (Harvard Apparatus, MA, USA). The abdominal aorta was isolated, and the catheter for DSI radiotelemetry was cannulated. A DSI data acquisition system was applied for continuous recording of mean arterial blood pressure (MAP).

For weekly monitoring of tail artery BP, SBP, diastolic blood pressure (DBP) and mean artery pressure (MAP) of tail artery were measured between 9:00 AM and 12:00 AM in conscious rats using a noninvasive computerized tail-cuff system (Kent Scientific Corporation, CT, USA). Daily BP measurements were performed for at least 1 week before experiments in order to minimize stress-induced tail artery BP fluctuations. Rats were warmed for 10–20 min at 28 °C before the measurements to allow for detection of tail arterial pulsations and to achieve a steady pulse. Tail artery BP was obtained by averaging 8–12 measurements as described previously^48^. To ensure the reliability of BP values by tail-cuff methods, we also compared the value between tail-cuff and radiotelemery methods in same rats at same points, and our data showed good consistency between these two methods (Supplementary Fig. 3a).

### Peripheral blood samples

For rat peripheral blood samples, periorbital puncture was performed with the 3.5% isoflurane-anesthetized rats resting on its left side. The eye was made to protrude by distending the facial skin with the thumb and index finger at the same time occluding the jugular vein. A haematocrit tube (BD Biosciences, CA, USA) was pushed while being gently rotated through the conjunctiva medially into the ocular cavity of the right eye. When blood rose in the tube, the jugular occlusion was terminated and the haematocrit tube removed. The eyelids were closed and gentle pressure applied for at least 10 s to minimize retro-orbital haemorrhage.

Blood samples were immediately fractionated, aliquoted into EP tubes (0.5 mL), and stored at −80 °C for examination. The plasma CGRP levels were determined by a commercially available ELISA kit (CEA876Hu. Cloud-Clone Corp., Wuhan, China) according to the manufacturer’s instructions.

### Perirenal adipose tissue ablation (PRATA)

Rats were anesthetized with isoflurane (3.5% for induction, 2.5% maintenance). An abdomen midline incision was made. And then the kidneys were exposed. The small artery suppling perirenal adipose tissue was clamped with vascular forceps to avoid bleeding. Then, bilateral perirenal adipose tissues away from the adrenal gland were carefully removed using an ophthalmic scissor. The pararenal, epididymal and inguinal adipose tissue ablation was made bilaterally similar to perirenal adipose tissue ablation procedure. After removal of adipose tissue, the viscera were then positioned back.

### Nerve activity recording

Renal sympathetic nerve activity (RSNA) was recorded for the dynamic changes of sympathetic outflow as previously reported^49,50^. Left RSN was isolated with the aid of an operating microscope. The renal nerve was cut distally to eliminate its afferent activity for RSNA recording, or proximally to eliminate its efferent activity for renal afferent nerve activity recording. Nerve was placed on a pair of silver electrodes and immersed in mineral oil. RSNA was amplified using an AC/DC differential amplifier (A-M System Inc., WA, USA) with a low-frequency cut-off at 60 Hz and a high-frequency cut-off at 3000 Hz. The amplified and filtered signals were integrated at the time constant of 100 ms and recorded by the PowerLab data acquisition system (AD Instruments, Sydney, Australia). The percentage change in integrated RSNA from the baseline value was calculated after PRATA. For cervical vagus nerve activity (CVNA) recording, a neck midline incision was made, and the left cervical vagus nerve was isolated for recording CVNA.

### Laser speckle contrast imaging

Local blood flow was measured using the Moor FLPI Full-field Laser Perfusion Imager (MoorFLPI, UK). The distance between laser head and tissues was 20–30 cm. Local blood flow values of 5 min before PRATA were measured and set as baseline, then the local blood flow values after PRATA were obtained every 10 min until 60 min as previously described^51^.

### Angiotensin II treatment

Male WKY rats weighing 250 g were infused with 200 ng/kg/min Ang II (Sigma, USA) via osmotic minipumps (ALZET model 2006, CA, USA) for 6 weeks. At the 2nd week after Ang II treatment, rats were divided into three groups receiving sham surgery, PRATA and pararenal adipose tissue ablation treatment. Then weekly BP measurement was performed for 4 weeks.

### Labeling of afferent nerves projecting into adipose tissues

For labeling of neuron soma that project into adipose tissue, male SHRs were anesthetized with isoflurane, then an incision was made on the abdominal midline to expose the abdominal cavity. Viscera that obstructed the perirenal and pararenal fat pad were reflected to expose the retroperitoneal space. DiI (1,1′-dioctadecy1–3,3,3′,3′-tetramethylindocarbocyanine perchlorate) or DiO (3,3′-dioctadecyloxacarbocyanine perchlorate) was diluted into 1 mmol/L with dimethyl sulfoxide. Approximately 50 μL per side of DiI was injected into perirenal adipose tissue bilaterally using a 25-gauge needle (5.0 μL per site, 10 sites for each side). The same dose of DiO was bilaterally injected into pararenal adipose tissue. The fat pads were inspected for any leakage to surrounding internal organs, and rinsed with sterile 0.9% saline. The viscera were then repositioned back. The abdominal muscles and skin were closed with sutures.

For labeling of neural fibers inside adipose tissues, slides of paraffin-embedded adipose tissues were processed for immunofluorescence staining. After dewaxing and hydration, the tissue slides were rinsed with PBS and blocked for 1 h with 2.5% bovine serum albumin. Adipose tissue slides were incubated with pan-axonal antibodies (SMI-312, BioLegend, CA, USA) and rabbit anti-CGRP (1:100; #14959, CST, MA, USA) for 24 h. After incubation with the primary antibody, the tissue slides were rinsed with PBS and incubated with Alexa Flour 488-conjugated anti-rabbit secondary antibody (Jackson Immunoresearch, PA, USA; at a dilution of 1:200) and Cy3-conjugated anti-mouse species secondary antibody (Jackson Immunoresearch; at a dilution of 1:200; for SMI-312) for 1 h. Then, the tissue slides were rinsed with PBS and incubated in FluoroGold with DAPI (Invitrogen) and images were captured.

### Dorsal root ganglia (DRG) injection

The bilaterally direct DRG injections were performed as previously described^52^. For right-side DRG injection, rats were placed in a prone position under isoflurane anesthesia. An ~4 cm longitudinal incision was made in skin just to the right of the dorsal midline starting from the costovertebral angle. After incising the superficial muscular fascia, paraspinal muscles were separated by a combination of sharp and blunt dissection, then exposing the lateral aspect of the thirteenth thoracic (T13) to third lumbar (L3) vertebrae and the dorsal aspect of the medial portion of their transverse processes. Before injection, foramen was slightly enlarged in a cephalad direction using rongeur to remove a 1 mm-deep crescent of laminar bone, exposing the distal third of the DRG. After exposing the DRG, a volume of 1 μL of capsaicin or AAV-EF1a-DIO-hM4Di-mCherry (9 × 10^9^ infectious units/μL) was slowly injected into the DRG using a 1 μL micro-syringe. When injection was completed, the needle was held on the DRG for 5 min to prevent leakage.

### Perirenal fat denervation by resiniferatoxin (RTX)

Resiniferatoxin (RTX, Sigma, MO, USA) was dissolved and stocked in absolute ethanol. RTX stock solution was diluted into a final concentration of 20.0 pmol/μL with the volume ratio (1% of the stock solution, 1% of Tween 80, and 98% of saline) before injection^18^. According to PRATA procedure, RTX or vehicle was bilaterally equably injected into perirenal or pararenal fat (for acute experiment: 8 μL per site, 4 sites for each fat pad; for chronic experiment: 1 μL per site, 5 sites for each fat pad) after kidney was exposed^18^.

### Tissue processing and immunofluorescence staining

Six groups of rats, including SHR sham (*n* = 10), SHR 4 weeks and 12 weeks post PRATA (*n* = 10 for each group) and WKY sham (*n* = 10), WKY 4 weeks and 12 weeks post PRATA (*n* = 10 for each group), were anesthetized with isoflurane and transcardially perfused with phosphate buffered saline (PBS) followed by 4% paraformaldehyde in PBS. The sections of spinal cord from thoracic (T) spinal cord level 13 (T13) and lumbar (L) spinal cord level 1 to 3 (L1-L3) were collected for cryostat sections (20 μm). The L1 and L2 DRGs were removed and processed for cryostat sections (10 μm). Samples were immunostained with primary polyclonal chicken anti-MAP2 (1:4000, NB300-213, Novus biological, CO, USA); monoclonal mouse anti-SMI312 (1:3000; #837904, Biolegend, CA, USA); polyclonal rabbit anti-NeuN (1:100; ab177487, abcam, MA, USA); rabbit anti-CGRP (1:100; #14959, CST, MA, USA); monoclonal mouse anti-c-Fos (1:100; ab208942, abcam, MA, USA); and rabbit anti-TRPV1 (1:100; ACC-030, alomone labs, Israel). For detection of primary antibodies, appropriate secondary antibodies, coupled to Alexa Flour 488 or Alexa Flour 594 (all from Jackson, PA, USA), were used. Specimens were examined with a confocal laser-scanning microscope (LSM710; Carl Zeiss Microimaging). The intensity of fluorescence per area was calculated by Image Pro plus 6.0 software (Media Cybernetics, MD, USA).

### Isolation of primary DRG neurons

SHRs were sacrificed with CO_2_ 4 weeks post PRAT ablation or surgery, and the fur was sprayed with 70% ethanol to prevent contamination. Bilateral DRG roots from L1 to L2 were harvested, and placed in 4 °C DMEM, then the nerve roots were trimmed. The DRGs were subjected to type I collagenase (3 mg/ml) treatment for 30 min at 37 °C, followed by 0.25% trypsin-EDTA for 40 min at 37 °C. The enzymatic reaction was stopped by DMEM containing 10% fetal bovine serum. Cells were passed through a 40 μm cell strainer, and were spun at 1200 rpm for 5 min. The supernatant was removed, and fresh growth medium was added. Then cells were plated on poly-D-lysine (0.1 mg/ml) treated culture dishes. Because of the difference in adhesion speed between DRGs and glial cells, after 5 h culture in incubator, we can flush the DRGs down. Then the DRGs were spun at 700 rpm for 5 min, the supernatant was removed, and Trizol (Invitrogen, USA) was added to suspend cells.

### Library construction and whole-transcriptome sequencing

Total RNA was extracted from DRG neurons using TRIzol (Invitrogen, USA) according to manufacturer’s instructions, followed by DNase I treatment to remove DNA contamination. Subsequently, total RNA was assessed by electrophoresis on a denaturing agarose gel and quantified by NanoDrop spectrophotometer (NanoDrop, USA).

For library preparation and RNA sequencing, 6 nanograms of RNA were used as input material and libraries were prepared by with the SMARTer Stranded Total RNA-Seq Kit (Takara Bio USA, Mountain View, CA, USA). In brief, samples were fragmented at 94 °C for 4 min prior to first-strand synthesis. Illumina adaptors and indexes were added to single-stranded cDNA via five cycles of PCR. Libraries were hybridized to R-probes for fragments originating from ribosomal RNA to be cleaved by ZapR. The resulting ribo-depleted library fragments were amplified with 13 cycles of PCR. Finally, the products were purified using the magicPure size selection DNA beads (EC401-03, Transgen Biotech. Co., China), and library quality was evaluated on an Agilent Bioanalyzer 2100 system. RNA library was sequenced on an Illumina Novaseq platform, and 150 bp paired-end reads were generated.

To analyze RNA sequencing data, clean reads were mapped to the reference genome by using HISAT2 v2.0.4. The mapped reads of each sample were assembled by using StringTie v1.3.1 in a reference-based approach^53^. The assembled transcripts were analyzed for coding potential by using RNA_seQc (http://www.broadinstitute.org/cancer/cga/rna-seqc). Fragments per kilobase of exon per million reads (FPKMs) for coding genes in each sample were calculated by using Cuffdiff v2.1.1^54^. Gene FPKMs were computed by summing FPKMs for transcripts in each gene group, then differentially expressed mRNAs were calculated by using the FPKM values for each gene.

### Transmission electron microscopy

L1 DRGs from WKY rats or SHRs were immersed in a solution of 2.5% glutaraldehyde overnight at 4 °C. After washing with phosphate buffer, DRGs were post-fixed in a 1% osmium acid for 2 h at 4 °C. After two washes with ddH2O, samples were stained in 2% uranyl acetate for 2 h, dehydrated in increasing concentration of ethanol, and infiltrated with 100% acetone for 2 h and EPON 812 resin overnight. Then, DRGs were polymerized at 37 °C for 12 h, 45 °C for 12 h and 60 °C for 48 h. Thin sections of 70 nm were obtained and analyzed on a transmission electron microscope (FEI Tecnai G2 Spirit Bio TWIN).

### Real-time PCR

L1-L2 DRGs dissected from SHR and WKY rats were pooled and processed for RNA extraction and reverse transcription within 1 h and were subjected to cDNA amplification and purification. DRGs were gently transferred into lysis buffer, and reverse transcription was performed using a SMARTer Ultra Low RNA Kit (Clontech) directly in the cell lysate. Single-cell cDNA was amplified using an Advantage 2 PCR Kit (Clontech) according to the manufacturer’s protocol. Real-time PCR was performed on an ABI Prism 7900 system. Taqman probes to detect CGRP and Actin were purchased from Roche. Each sample was analyzed in triplicate, and the relative levels of target genes were normalized to Actin. Fold changes were then calculated for each treatment group using normalized CT values. Taqman primers and probes used for the amplification were as follows: rat CGRP (sense primer, 5′-CCTCCAGGCAGTTCCTTTG-3′; anti-sense primer, 5′-CCAGTAGGCGAGCTTCTTCTT-3′; probe, Roche UPL #26), rat β-actin (sense primer, 5′- GCCCTAGACTTCGAGCAAGA-3′; anti-sense primer, 5′-GACCGTCAGGCAGCTCATAG-3′; probe, Roche UPL #80).

### Designer receptors exclusively activated by designer drugs, DREADDs

Rats were anesthetized with 1.5% isoflurane. The perirenal adipose tissue was exposed following PRATA procedure. Then, 1 μL of either control or retro-AAV2-Cre viral vector for each point was bilaterally injected into the perirenal adipose tissue, and five separated point injections for each side were performed. For DRG injection, when the perirenal adipose tissue injection was completed, rats were turned from supine to prone position. After exposing the intervertebral foramen leading to DRG, 1 μL of the AAV9-DIO-hM4Di-mcherry (EF1α promoter floxed) viral vector (~200,000 infectious units in 10% sucrose) was bilaterally infused into the L1-L2 DRGs. Three weeks post perirenal and DRG viral vector injection, rats received CNO (3 mg/kg) or vehicle 10 min prior to initiating BP data collection. Continuous intracarotid MAP measurements were recorded for 70 min.

### Measurement of serum CGRP and CGRP antagonist treatment

Rat blood samples were collected from eyeball and the serum was obtained by centrifugation at 1600 *rpm* (310 g) for 15 min and stored at −80 °C until analysis. Serum CGRP concentration was determined using a commercially available ELISA kit (KE1754, ImmunoWay Biotechnology, TX, USA) according to the manufacturer’s instructions. For acute experiments, 1 mg/kg CGRP antagonist, CGRP8-37 (Sigma, MO, USA), was intravenously injected 5 min after PRATA, and continuous intracarotid BP were recorded for 70 min. For chronic experiments, CGRP8-37 treatment was performed one week after PRATA or sham operation. All rats received the once daily intraperitoneal injection of 10 μg/kg CGRP8-37 or saline for 4 weeks^55^. Tail artery BP was measured weekly for 6 weeks. CGRP8-37 was freshly dissolved in saline before daily injection.

### Catecholamines and related metabolites measurement

Blood samples were collected from eyeball and transferred into tubes containing EDTA, and plasma was obtained by centrifugation at 10,000 *g* at 4 °C for 15 min and stored at −80 °C until analysis. Twenty-four hour-urine samples were collected from the rats housed in metabolic cages (TSE Systems, Thuringia, Germany) and fixed with concentrated hydrochloric acid (10.4 M) and the examination was completed within 12 h. Both catecholamines and related metabolites from plasma or urine were measured by high performance liquid chromatography, HPLC in The First Affiliated Hospital of Nanjing Medical University.

### Statistical analysis

All data were expressed as mean ± standard error of the mean (SEM) or median (interquartile range). Sample size for each experiment was calculated by analyzing preliminary-experimental data with the PASS statistical software (NCSS, UT, USA). Data were graphed using GraphPad Prism 7.0 (GraphPad Software, San Diego, CA, USA). Differences between two groups were analyzed with unpaired t test, Mann–Whitney U test or paired Wilcoxon signed-rank test. One-Way analysis of variance (ANOVA) with Bonferroni post hoc test analysis was used for comparison among groups. *P* values < 0.05 were considered statistically significant.

### Reporting summary

Further information on research design is available in the Nature Research Reporting Summary linked to this article.

## Supplementary information

Supplementary Information

Reporting Summary

## Data Availability

The RNA-seq data generated in this study have been deposited in the NCBI Gene Expression Omnibus (GEO) database under accession code GSE160914. All other data generated or analyzed during this study are included in this published paper (and its Supplementary Information files). Additional data related to this paper are available from the corresponding author on reasonable request. Source data are provided with this paper.

## Source data

Source Data
