# Supplementary material for: Perirenal adipose afferent nerves sustain pathological high blood pressure in rats
Source: Nat Commun. 2022 Jun 6;13:3130. doi: 10.1038/s41467-022-30868-6 (PMC9170717; doi:10.1038/s41467-022-30868-6)
Supplement: Supplementary file 1 — Supplementary Information [file 41467_2022_30868_MOESM1_ESM.pdf]

Supplementary Material

Supplementary Methods

Anatomy for adipose tissue ablation and denervation

Details of the anatomical properties are discussed in our previous work [1, 2]. In brief, rats were anesthetized with isoflurane (3.5% for induction, 2.5% maintenance). An abdomen midline incision was made. And then the kidneys were exposed. Perirenal fat is within Gerota's fascia, while pararenal fat is outside Gerota's fascia. Perirenal fat ablation is done with care to ensure that adipose tissue outside Gerota's fascia remain intact. Conversely, when ablating pararenal fat, Gerota's fascia should not be dissected. (**Supplementary Fig. 1a and 1b**)

Radiotelemetry Implantation

To compare the tail-cuff method with radiotelemetry, which is considered as the gold standard of rat blood pressure (BP) measurement, 8 male spontaneously hypertensive rats (SHRs) were anesthetized with isoflurane (3.5% for induction, 2.5% maintenance). An abdomen midline incision was made. The skin was undermined for at least 1 cm around the edges of incision. Then the abdominal aorta were exposed, and the catheter of the radiotelemetry implant (PA-C10, Data Sciences International, New Brighton, MN) was cannulated. Tissue adhesive (Vetbond, 3M; St. Paul, MN) was used to avoid bleeding and to prevent the catheter from falling out. The body of the implant was secured in the abdominal subcutaneous tissue with six to eight interrupted nonabsorbable sutures (5-0 braided polyester). The skin was then closed with nonabsorbable sutures (3-0 braided polyester), and

topical antiseptic was applied.

Transcriptome Analysis

To determine potential roles of differentially expressed genes, GO and KEGG enrichment analyses were used with the clusterProfiler R-language package (http://bioconductor.org/packages/release/bioc/html/clusterProfiler.html).

For differential expressed gene abundances, we first generated their corresponding ENSEMBL ids [3]. Next, the ENSEMBL ids of differential expressed genes were fed into g: Profile [4], a tool to carry out functional pathway enrichment analysis. A functional enrichment adjusted P< 0.05 was treated as significant.

Spinal cord mutilation

The procedures were based on the method of a previous study[5]. Animals were anesthetized with isoflurane (3.5% for induction, 2.5% maintenance). All surgical procedures were performed under a microscope and under sterile conditions. After checking of the lowest lumbar level (L5), all rats underwent a left hemilaminectomy from vertebrae T12 to L3. Two drops of 1% lidocaine without epinephrine onto the spinal cord to prevent unexpected reflex movement during hemilaminectomy and after opening the dura mater. Then identify the left T12 DRG, and perform a complete cordotomy at the vertebra T12 level by cutting off the spinal cord using microscissors.

Mass Spectrometry

Products, reagents and instruments

Information of standard products and internal standards is shown in **Supplementary Table 1**.

Table S1 Information of standard products and internal standards

No.	Reagent	CAS	Manufacturer	Art.No
1	Adrenalin	51-43-4	TRC	E588580
2	Arterenol	55-27-6	TRC	N674500
3	Dopamine	62-31-7	TRC	D533780
4	Homovanillic acid	306-08-1	TRC	H669500
5	Vanillylmandelic acid	55-10-7	TRC	H946330
6	Creatinine	60-27-5	TRC	C781500
7	Adrenalin-d3	1217733-17-9	TRC	E588583
8	Noradrenaline-d6	/	Sigma	N-069
9	Dopamine-d4	203633-19-6	TRC	D533782
10	Homovanillic acid-d3	74495-71-9	TRC	H669502
11	Vanilla based tonsate-d3	74495-70-8	TRC	H946332
12	Creatinine-d3	143827-20-7	TRC	C781502
13	Angiotensin II	/	Anaspec	AS-20634
14	Angiiotensin-13C6,15N	/	Anaspec	AS-64805

Mass spectrometry (MS) grade methanol, acetonitrile, and HPLC grade ethylene acetate

were purchased from Fisher (USA). HPLC grade methanol and acetonitrile were purchased from Honeywell (USA). MS grade formic acid, MS grade methyl acid, benzyl sulfyl fluoride (PMSF), EDTA, 8-hydroxyquinoline sulfate were purchased from Merck (USA). Experimental water was prepared by PureLab Classic UVF (ELGA LabWater, UK) (resistivity = 18.2 MΩ).The 0.22 μm nylon filter was purchased from Agger. WCX 96 hole μElution plates and HLB 96 hole μElution plates were purchased from Waters. MS grade pure water as ultramorphic water was purchased from Fisher.

Qlife Lab 9000plus liquid chromatography-triple quadrupole tandem mass spectrometer was the main workstation for MS. MassHunter Workstation Data Acquisition color spectrum workstation was used as the chromatographic workstation. Other important instruments include METTLER Electronic Balance (XSR205); SCILOGEX D2012 high-speed desktop centrifuge; Vortex genie2 oscillator (USA);

Chromatographic condition

Flow phase A: 0.1% formic acid-water

Flow phase B: acetonitrile

Flow speed: 0.3 mL/min

Column temperature: 50°C

Column: Agilent Pursuit PFP (2.0×150 mm, 3.0 μm)

Table S2 Gradient elution conditions

Time (min)	Flow(mL/ min)	A (%)	B (%)
0.0	0.3	100	0
3.0	0.3	70	30

4.0	0.3	2	98
5.0	0.3	2	98
5.1	0.3	100	0
8.0	0.3	100	0

Mass spectrometry conditions

For parameters of MS, please refer to **Supplementary Table 3 and 4**.

Table S3 Parameters of MS ion sources

Project	Parameter
Capillary (V)	3500（ESI+）
Nozzle Voltage (V)	500（ESI+）
Gas Temp (C)°	350
Gas Flow (L/min)	7
Nebulizer (psi)	45
Sheath Gas Temp (C)°	400 (catecholamine), 350 (AngII)
Sheath Gas Flow (L/min)	11

Table S4 Parameters of ion-pairs

Chemical compound	Parent ion	Daughter ion	Fragmentor	Collision voltage (V)
E	184.1	107.1	60	21
NE	152	107	116	16
DA	154	137	60	5

HVA	181.1	122	80	11
VMA	181.1	149.1	72	13
Cre	114	86	90	10
E-d3	169	77.1	124	41
NE-d6	158	139.1	102	13
DA-d4	158.1	141.1	65	9
HVA-d3	184.1	140	72	5
VMA-d3	184.1	149	55	13
Cre -d3	117	89	73	10
AngII	523.9	784.4	110	18
AngII-13C6,15N	527.3	791.4	105	18

Preparation of standard solutions (SS) and standard curves

For catecholamine assays, the component of S0 solution is shown in **Supplementary Table 5**. Then SS in seven different concentrations were prepared using a serial dilution (E: 0.5,1,2,5,10,25, and 50 ng/mL; NE: 2,4,8,20,40,100, and 200 ng/mL. DA: 10,20,40,100, 200,500, and 1000 ng/mL. HVA and VMA: 100,200,400,1000,2000,5000, and 10000 ng/mL.

For Ang II assays, add 30 μL 40 ng/mL AngII SS into 1170 μL Tris solution (pH 6.0) containing 1%BSA. Then 1000 pg/mL AngII SS was obtained. Then SS in the next five concentrations (20,40,100,200,500 pg/mL) were prepared using a serial dilution.

Table S5 SS of catecholamine

Component	Master liquid concentration (μg/mL)	Master liquid volume (μL)	Bulk volume (μL)	S0 concentration (μg/mL)
E	100	10	1000	1
NE	400	10	1000	4
DA	2000	10	1000	20
HVA	5000	40	1000	200
VMA	5000	40	1000	200
Cre	20000	100	1000	2000

Preparation of working internal standard solution (ISS)

Press the table, a certain volume of catecholamine ISS is accurately added into 80% methanol solution of 9310μL to fully mix 10 mL working ISS following the recipe shown in **Supplementary Table 6**.

Table S6 Working ISS for catecholamine assays

Component	Master liquid concentration (μg/mL)	Move the volume (μL)	Bulk volume (μL)	Working ISS Concentration (ng/mL)
E-d3	10	40	10000	40
NE-d6	10	200	10000	200
DA-d4	100	20	10000	200

HVA -d3	100	400	10000	4000
VMA -d3	1000	10	10000	1000
Cre -d3	5000	20	10000	10000

Preparation of the standard curves for angiotensin II

Add 30 μL 40 ng/mL AngII standard solution to 1170 μL Tris solution (pH 6.0) containing 1%BSA as the first high value concentration point. The second highest concentration point was diluted with 350 μL Tris solution containing 1%BSA (pH 6.0). The first high value concentration point was diluted with 4 times volume Tris solution containing 1%BSA (pH 6.0) to get the third high value concentration point. The second highest concentration point was diluted with 4 times volume Tris solution containing 1%BSA (pH 6.0) to obtain the fourth highest concentration point. The third highest concentration point was diluted with 4 times volume Tris solution (pH 6.0) containing 1%BSA to get the fifth highest concentration point. The fourth highest concentration point was diluted with 4 times volume Tris solution containing 1%BSA (pH 6.0) to get the sixth highest concentration point.

Pre-treatment of samples

For catecholamine assay, 150 μL urine of the sample to be tested was passed through 0.22 μm filter membrane, 50 μL filtrate was put into a labeled 1.5 mL EP tube, 20 μL mixed internal standard working solution was added, 200 μL 10% methanol containing 0.1% formic acid was added, vortex mixing was carried out for 5s, 100 μL supernatant was taken for sampling.

For Ang II assay, kidney homogenate or calibration samples were taken with 125μL plus

25μL of enzyme inhibitors (containing 1 mM PMSF, 20 mM 8-hydroxyquinoline, and 250 mM EDTA of Tris solution), Add 150 L (AngII-13C6, 15N 0.1ng /mL), Vortex mixing well, Then 250μL was added to HLB SPE plates activated with methanol and 5% formic acid, After applying to the upper sample, Wash with 200μL 5% formic acid added to the SPE plate, They was then washed with 200 μL 20% methanol, Finally, it was eluted with 100μL of methanol, Collect elate for samples.

Statistics and Reproducibility

The experiments for Figure 5a and Supplementary Figures 1c; 5a; 6b; 7a, d; 8; 9a, e; 11e, g were repeated for three times independently with similar results

References:

[1]. Liu, B.X., et al., Distribution, Morphological Characterization, and Resiniferatoxin-Susceptibility of Sensory Neurons That Innervate Rat Perirenal Adipose Tissue. Front Neuroanat, 2019. 13: p. 29.
[2]. Liu, B.X., W. Sun and X.Q. Kong, Perirenal Fat: A Unique Fat Pad and Potential Target for Cardiovascular Disease. Angiology, 2019. 70(7): p. 584-593.
[3]. Zerbino, D.R., et al., Ensembl 2018. Nucleic Acids Res, 2018. 46(D1): p. D754-D761.
[4]. Raudvere, U., et al., g:Profiler: a web server for functional enrichment analysis and conversions of gene lists (2019 update). Nucleic Acids Res, 2019. 47(W1): p. W191-W198.
[5]. Dam-Hieu, P., et al., Regeneration of primary sensory axons into the adult rat spinal cord via a peripheral nerve graft bridging the lumbar dorsal roots to the dorsal column. J Neurosci Res, 2002. 68(3): p. 293-304.

Supplementary Figures

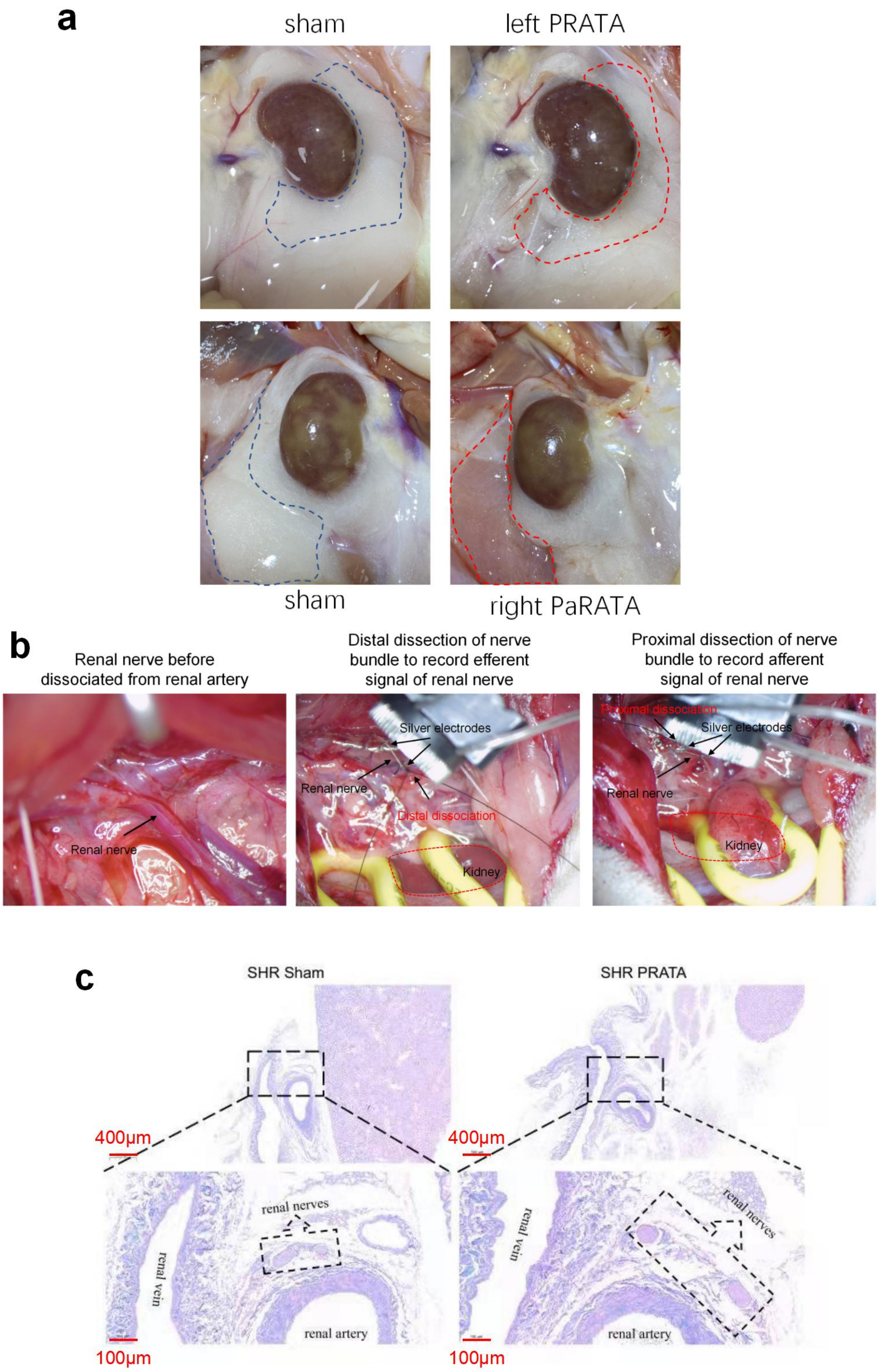

Figure S1. Illustration of anatomy and surgical techniques of PRAT

(**a**) Illustration of PRATA and PaRATA. Notice the location and range (blue dashed line for pre-operative, red dashed line for post-operative) of PRAT and PaRAT. (**b**) Illustration of the three core steps of renal nerve activity recording. (**c**) Luxol Fast Blue staining showed no obvious Wallerian degeneration in PRATA group compared with sham group.

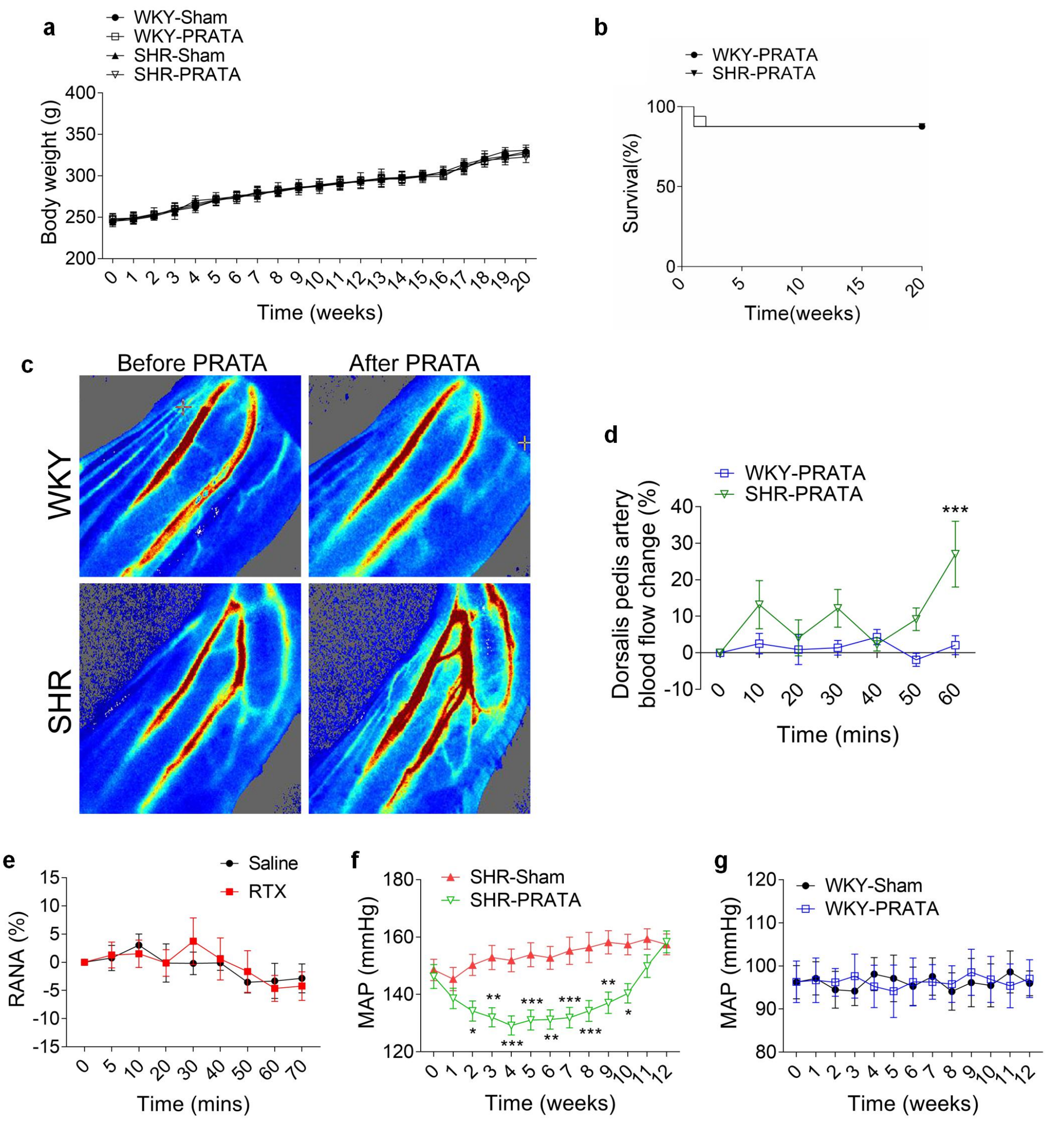

Figure S2. Various acute and chronic effects of PRATA

(**a**) No significant difference was discovered among PRATA groups and sham groups in weekly measurement of body weight, n=6 per group; Data are mean ± SEM. *P*>0.05. ANOVA, Bonferroni post-hoc test, two-sided. (**b**) No significant difference was discovered between survival rates of WKY rats and SHRs after PRATA, n=14 per group; *P*>0.05 Logrank test, two-sided. (**c** and **d**) Laser speckle contrast imaging of dorsalis pedis artery post PRATA and quantification, n=5 per group; Data are mean ± SEM. ****P*<0.001. ANOVA, Bonferroni post-hoc test, two-sided. (**e**) Renal afferent nerve activity (RANA) after RTX

injection into PRAT, n=6 for saline group, n=7 for RTX group, $P>0.05$. ANOVA, Bonferroni post-hoc test, two-sided. (**f** and **g**) Weekly measurement of MAP after PRATA using tail-cuff in female SHRs (**f**) and WKY rats (**g**), n=8 per group for SHRs, n=7 per group for WKY rats; *$P<0.05$, **$P<0.01$, ***$P<0.001$. ANOVA, Bonferroni post-hoc test, two-sided.

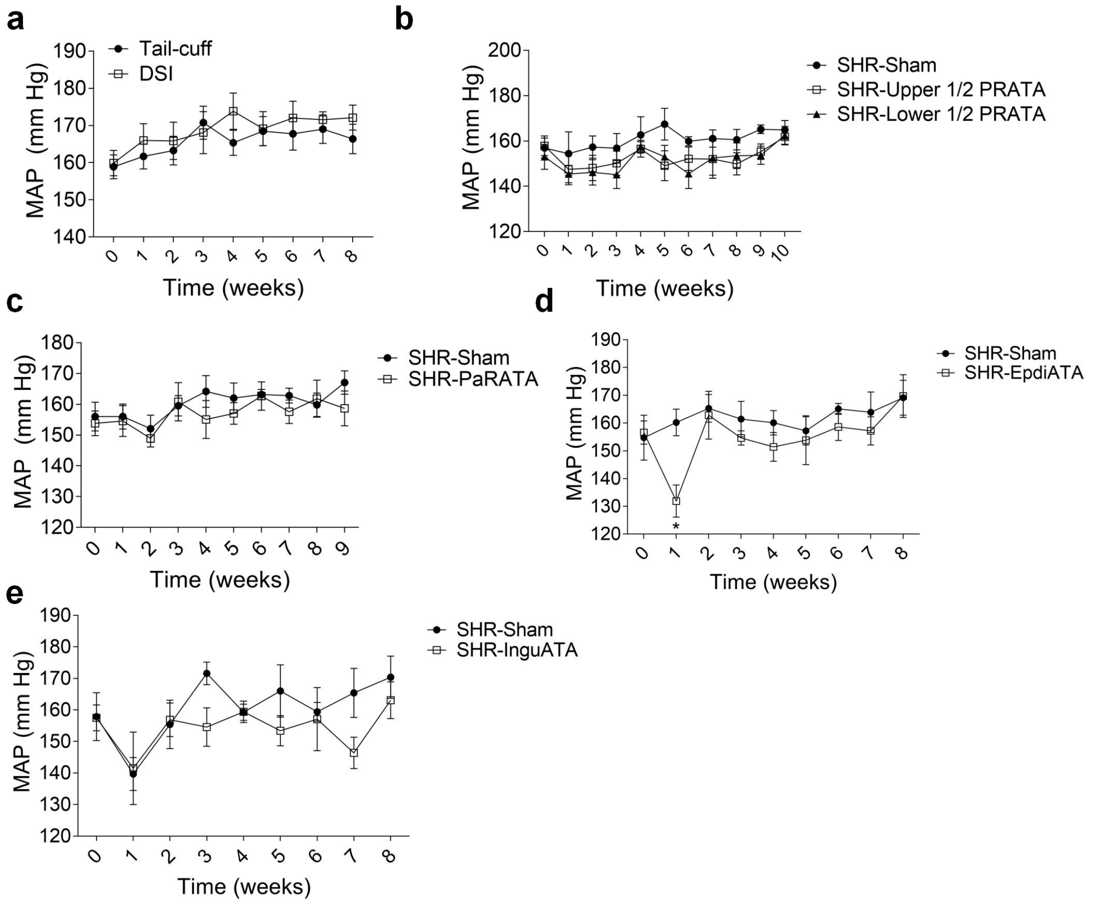

Figure S3. Chronic effects of various fat ablation strategies on BP

(**a**) Comparison of arterial BP of SHRs using tail-cuff and radiotelemetry (DSI) in daytime, n=8, per group; Data are mean ± SEM. *P*>0.05. ANOVA, Bonferroni post-hoc test, two-sided. (**b**) Weekly measurement of tail arterial BP after half-volume PRATA, n=6, per group; Data are mean ± SEM. *P*>0.05. ANOVA, Bonferroni post-hoc test, two-sided. (**c**) Weekly measurement of tail arterial BP after pararenal adipose tissue ablation in SHRs, n=7, per group; Data are mean ± SEM. *P*>0.05. ANOVA, Bonferroni post-hoc test, two-sided. (**d**) Weekly measurement of tail arterial BP after epididymal fat ablation in SHRs, n=6, per group; Data are mean ± SEM. **P*<0.05. ANOVA, Bonferroni post-hoc test, two-sided. (**e**) Weekly measurement of tail arterial BP after inguinal fat ablation in SHRs, n=6, per group; Data are mean ± SEM. *P*>0.05. ANOVA, Bonferroni post-hoc test, two-sided. PaRATA, pararenal

adipose tissue ablation; EpdiATA, epididymal adipose tissue ablation; InguATA, inguinal adipose tissue ablation.

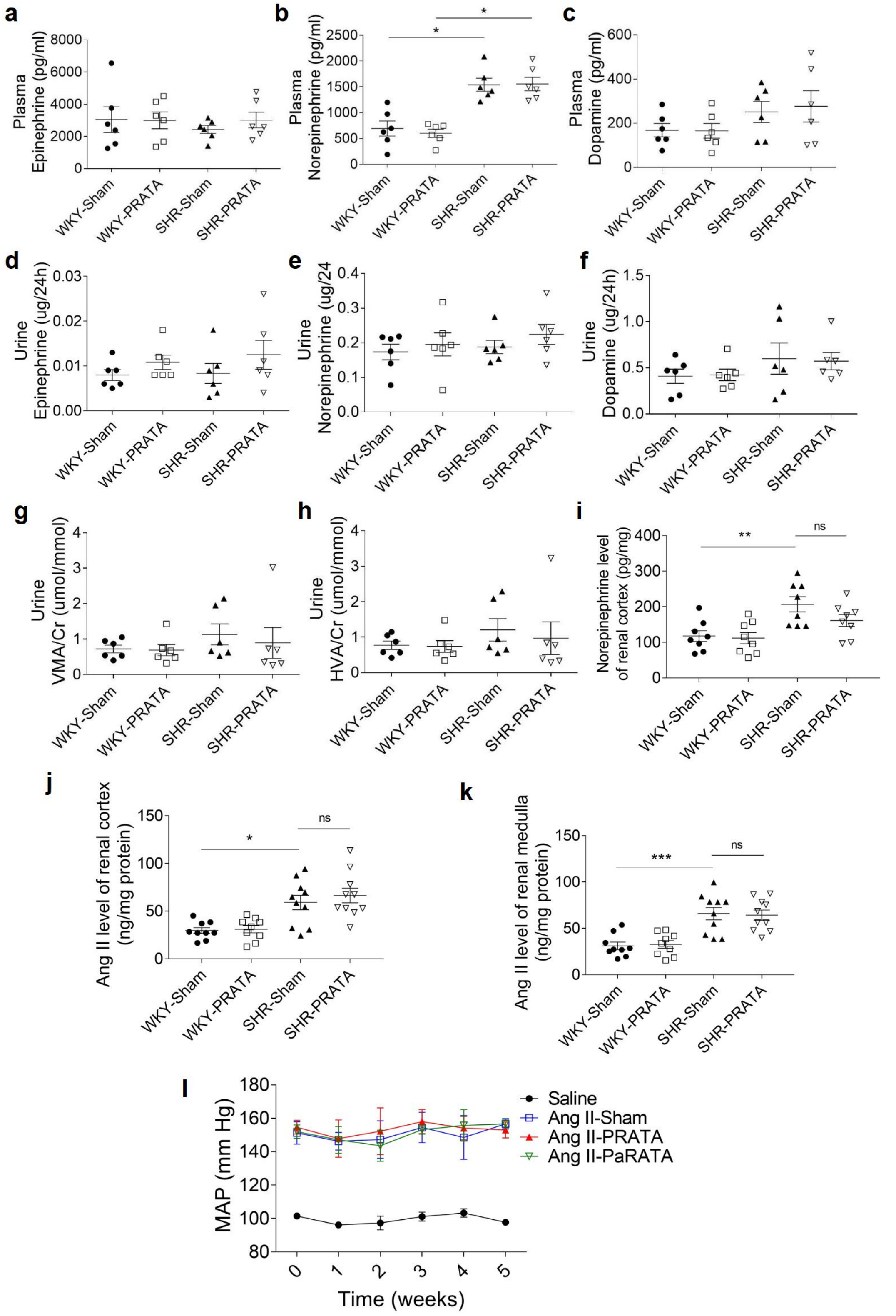

Figure S4. Levels of catecholamine and angiotensin II in plasma, urine and kidneys at 4 weeks after PRATA

(**a** to **c**) Plasma levels of epinephrine (a), norepinephrine (b) and dopamine (c) were

measured by HPLC; n=6 per group, Data are mean ± SEM. *$P<0.05$. ANOVA, Bonferroni post-hoc test, two-sided. (**d** to **f**) Urine levels of epinephrine (d), norepinephrine (e) and dopamine (f) were measured by HPLC; n=6 per group, Data are mean ± SEM. $P>0.05$. ANOVA, Bonferroni post-hoc test, two-sided. (**g** and **h**) relative urine levels of VMA (g) and HVA (h); n=6 per group, Data are mean ± SEM. $P>0.05$. ANOVA, Bonferroni post-hoc test, two-sided. (**i**) Levels of norepinephrine in renal cortex measured by ELISA; n=8 per group, Data are mean ± SEM. **$P<0.01$, ns $P>0.05$. ANOVA, Bonferroni post-hoc test, two-sided. (**j** and **k**) Levels of angiotensin II (Ang II) in renal cortex or medulla measured by mass spectrometry; n=9 for WKY-Sham and WKY-PRATA groups, n=10 for SHR-Sham and SHR-PRATA groups, Data are mean ± SEM. *$P<0.05$, ***$P<0.001$. ANOVA, Bonferroni post-hoc test, two-sided. (**l**)Weekly measurement of tail arterial BP after PRATA and PaRATA in Ang II-treated and control WKY rats, n=6 per group; Data are mean ± SEM. $P>0.05$. ANOVA, Bonferroni post-hoc test, two-sided.

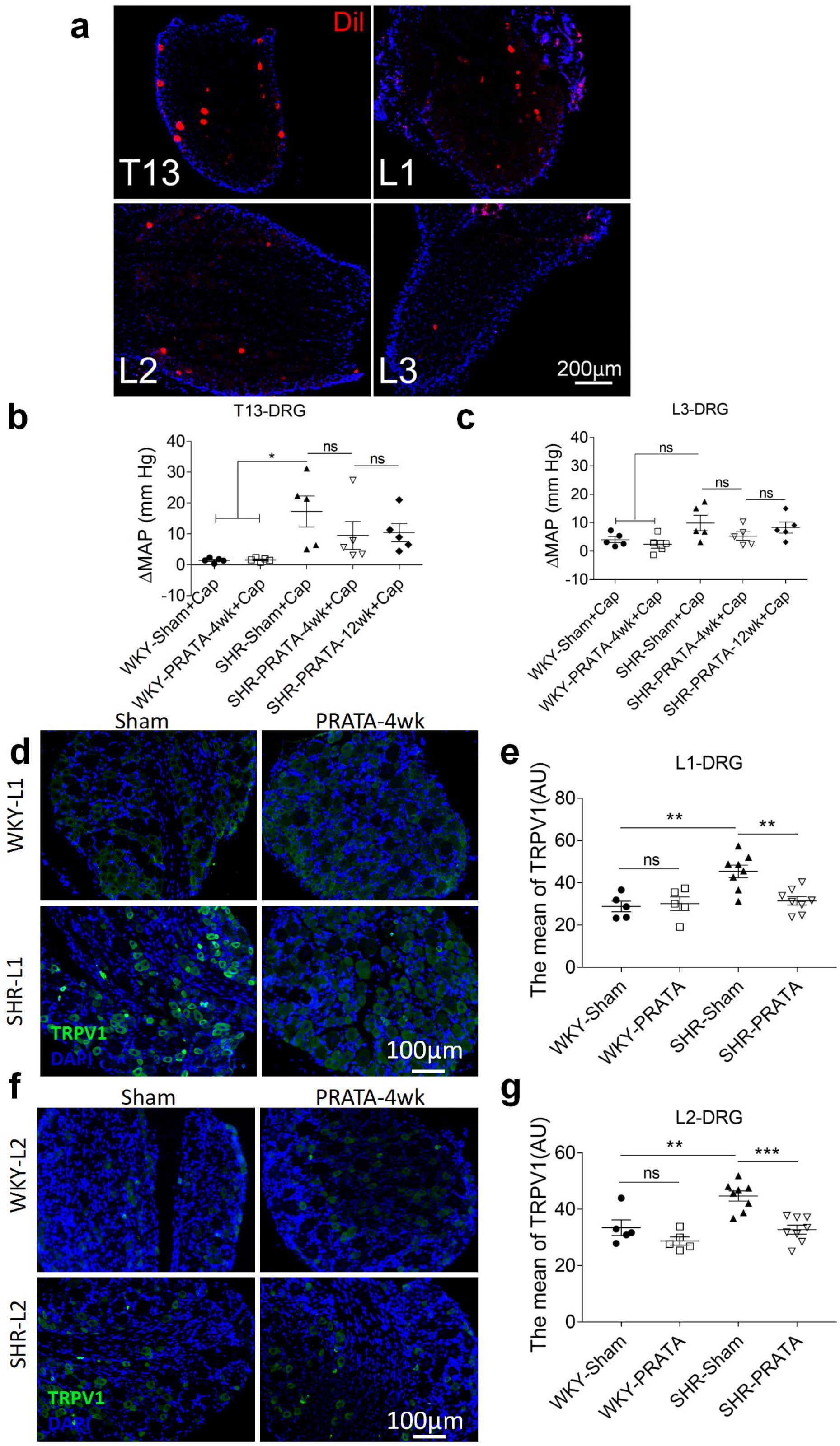

Figure S5. Retro-labeling and activation of DRG neurons projecting to PRAT

(a) DiI-labelled neurons of T13-L3 DRG after PRAT DiI injection in SHR rats. **(b and c)** Changes of intra carotid arterial MAP under T13 and L3 DRG injection with capsaicin post

PRATA, n=5 per group; Data are mean ± SEM, P>0.05 non-significant (ns), *P<0.05. ANOVA, Bonferroni post-hoc test, two-sided. (**d-g**) Immunostaining of TRPV1 in L1 (**d**) and L2 (**f**) DRGs after PRATA and quantification (**e** and **g**), n=5 for WKY-Sham and WKY-PRATA groups, n=8 for SHR-Sham and SHR-PRATA groups; **P<0.01, ***P<0.001. ANOVA, Bonferroni post-hoc test, two-sided. AU, arbitrary unit.

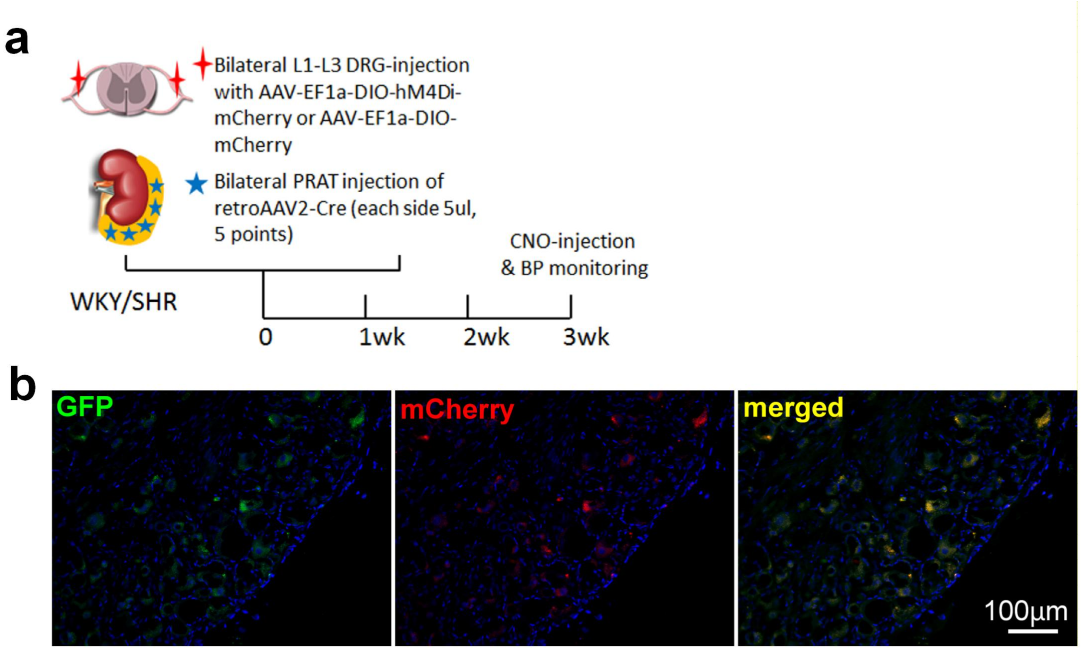

Figure S6. Regulation of DRG neurons by Gi-DREADD strategy

(**a**) Illustration of chemogenetic manipulation of sensory neurons using Gi-DREADD in SHR and WKY rats. (**b**) Representative imaging of transfected neurons in L1 DRG.

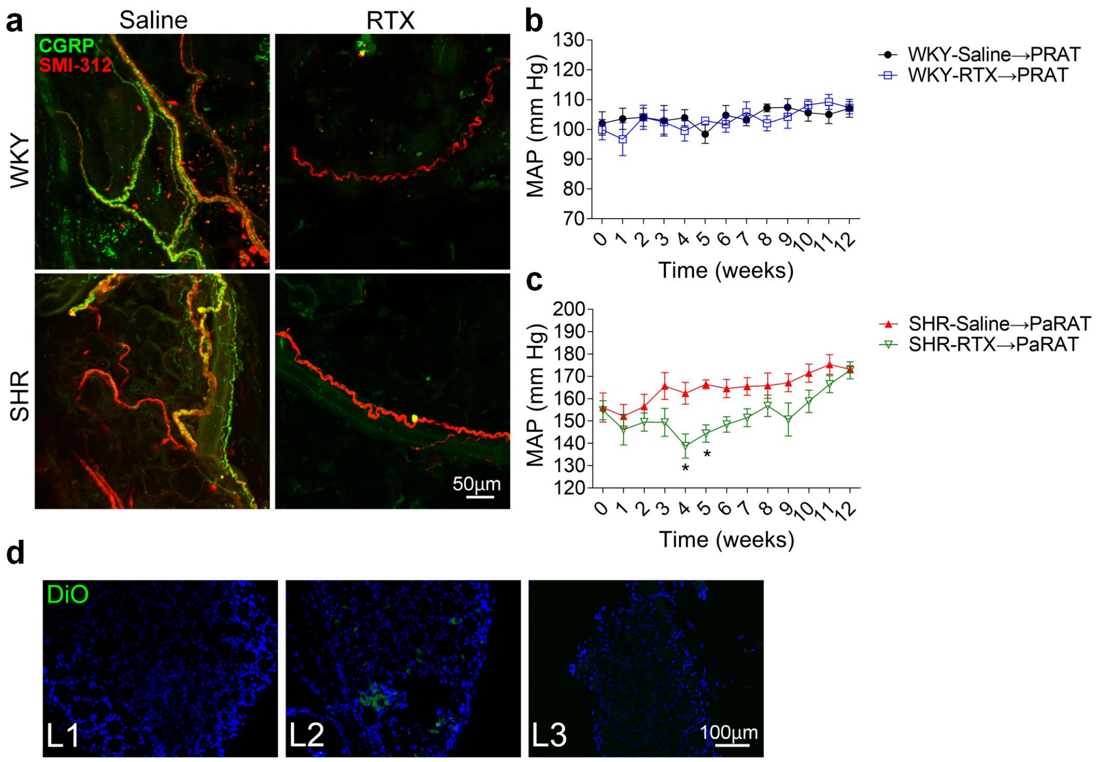

Figure S7. PRATD and PaRAT denervation by RTX

(**a**) Immunostaining of SMI-312 and CGRP in PRAT without (left) or with (right) RTX injection. (**b**) Weekly measurement of tail arterial BP post PRAT denervation by RTX in WKY rats, n=6 per group; Data are mean ± SEM, $P>0.05$. ANOVA, Bonferroni post-hoc test, two-sided. (**c**) Weekly measurement of tail arterial BP post PaRAT denervation by RTX in SHR rats, n=6 per group; Data are mean ± SEM, $^*P<0.05$. ANOVA, Bonferroni post-hoc test, two-sided. (**d**) DiO-labeled neurons in L1-L3 DRGs after injecting DiO into PaRAT in SHRs. PaRAT, pararenal adipose tissue

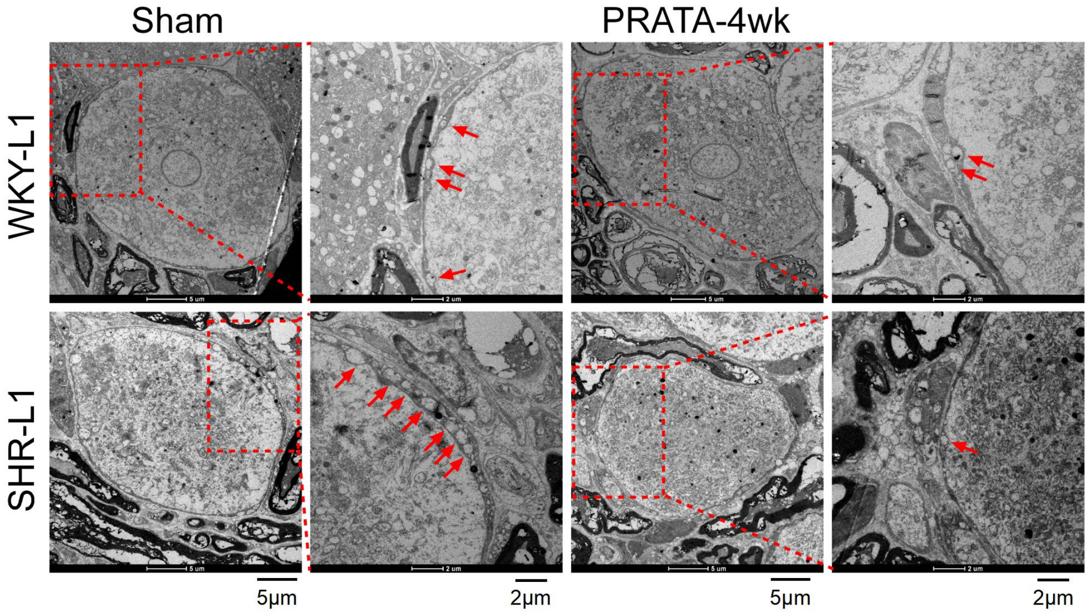

Figure S8. Ultrastructure of L1 DRG neuron after PRATA

Representative imaging of L1 DRG neurons from WKY rats and SHRs, arrows indicating the microtubules of neuron surface reflecting the base of dendritic shape morphology.

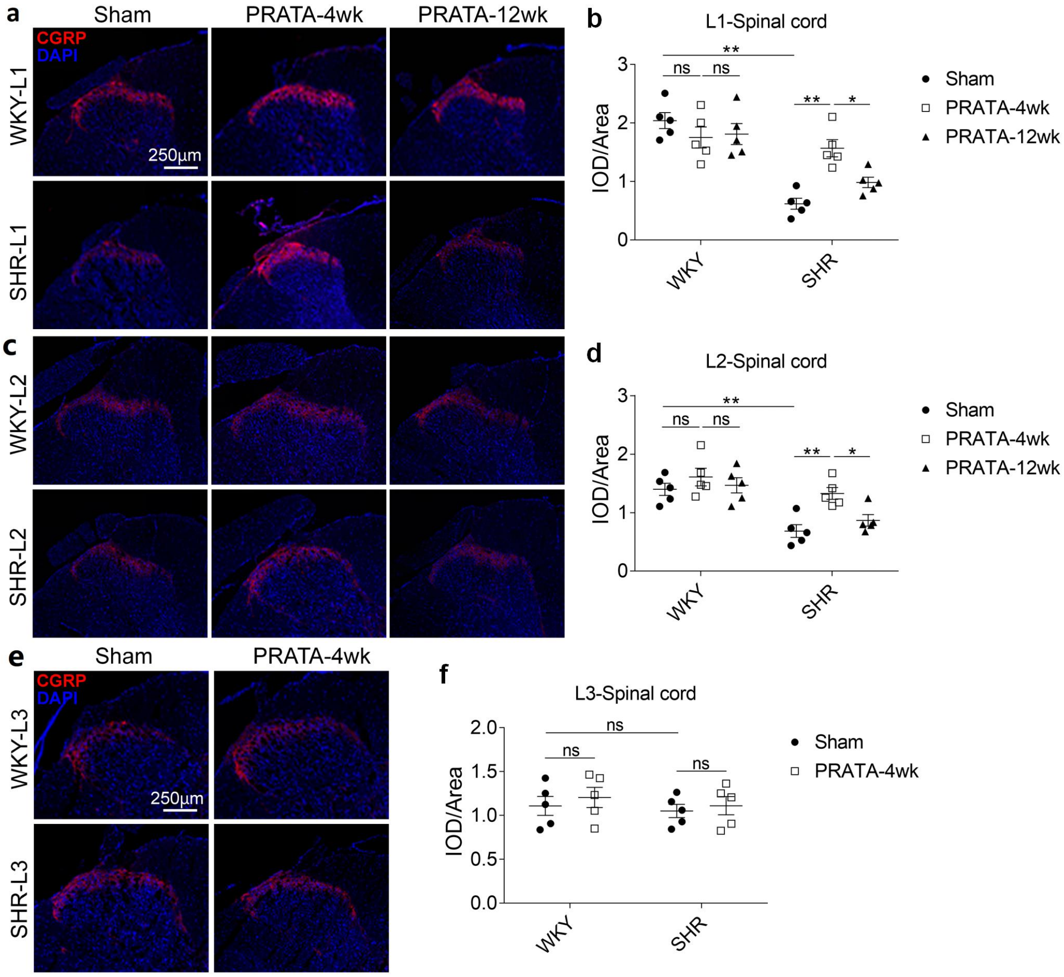

Figure S9. Levels of CGRP in L1-L3 spinal cord after PRATA/PRATD

(**a** to **d**) Immunostaining of CGRP in L1-L2 spinal cord after PRATA (**a**, **c**), and quantification of fluorescence intensity (IOD/Area) (**b**, **d**), n=5 per group; Data are mean ± SEM, **P*<0.05, ***P*<0.01. ANOVA, Bonferroni post-hoc test, two-sided. (**e**, **f**) Immunostaining of CGRP in L3 spinal cord after PRATD (e), and quantification of fluorescence intensity (IOD/Area) (f), n=5 per group; Data are mean ± SEM, *P*>0.05. ANOVA, Bonferroni post-hoc test, two-sided.

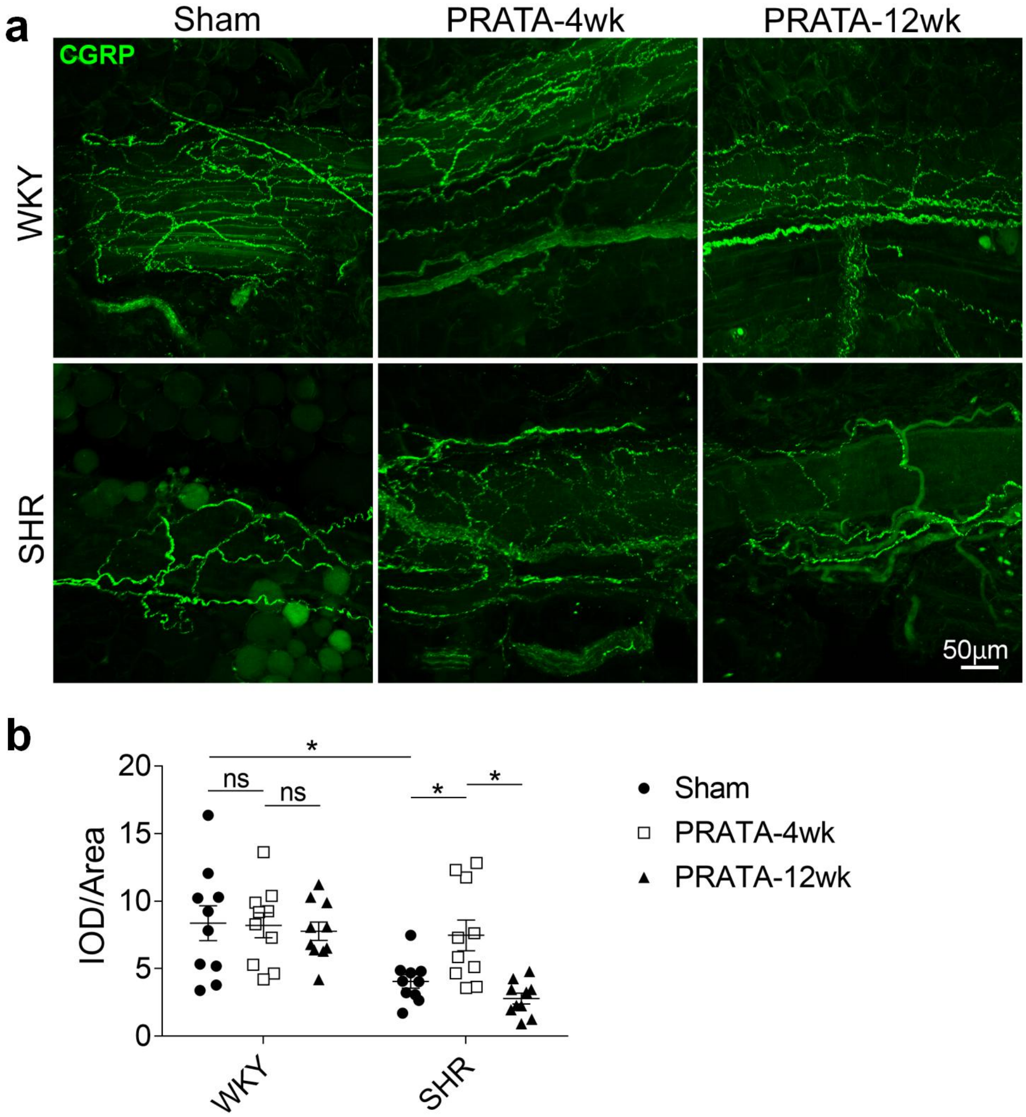

Figure S10. Levels of CGRP in mesentery artery after PRATA

(**a** and **b**) Immunostaining of CGRP in mesentery arteries before and after PRATA (a), and quantification (b), n=10 per group; Data are mean ± SEM, *P*>0.05 non-significant (ns), *$P<0.05$. ANOVA, Bonferroni post-hoc test, two-sided.

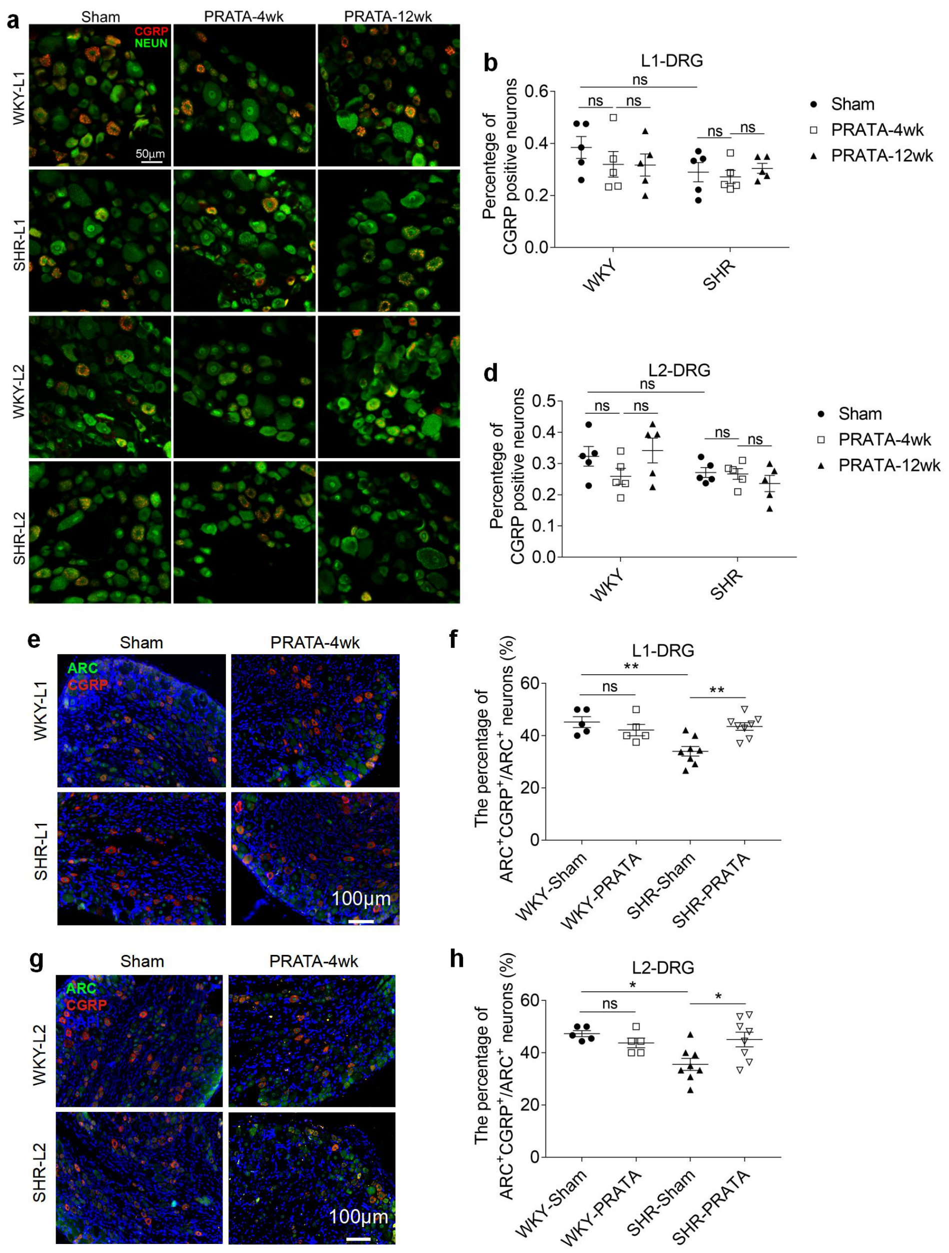

Figure S11. Levels of CGRP in L1-L2 DRG neurons

(**a** to **d**) Immunostaining of CGRP in L1-L2 DRGs before and after PRATA (a, c), and quantification (b, d), n=5 per group; Data are mean ± SEM, P>0.05 non-significant (ns). ANOVA, Bonferroni post-hoc test, two-sided. (**e** to **h**) Immunostaining of CGRP and ARC in L1-L2 DRG after PRATD. Data are mean ± SEM, *P<0.05, **P<0.01. ANOVA, Bonferroni post-hoc test, n=5 for WKY-Sham and WKY-PRATA group, n=8 for SHR-Sham and SHR-PRATA group, two-sided.
