# Supplementary material for: Perirenal adipose afferent nerves sustain pathological high blood pressure in rats
Source: Nat Commun. 2022 Jun 6;13:3130. doi: 10.1038/s41467-022-30868-6 (PMC9170717; doi:10.1038/s41467-022-30868-6)
Supplement: Supplementary file 2 — Reporting Summary [file 41467_2022_30868_MOESM2_ESM.pdf]

nature portfolio

Corresponding author(s): Wei Sun, Xiangqing Kong

Last updated by author(s): Mar 29, 2022

Reporting Summary

Nature Portfolio wishes to improve the reproducibility of the work that we publish. This form provides structure for consistency and transparency in reporting. For further information on Nature Portfolio policies, see our Editorial Policies and the Editorial Policy Checklist.

Statistics

For all statistical analyses, confirm that the following items are present in the figure legend, table legend, main text, or Methods section.

n/a	Confirmed	
☐	☒	The exact sample size (*n*) for each experimental group/condition, given as a discrete number and unit of measurement
☐	☒	A statement on whether measurements were taken from distinct samples or whether the same sample was measured repeatedly
☐	☒	The statistical test(s) used AND whether they are one- or two-sided<br>*Only common tests should be described solely by name; describe more complex techniques in the Methods section.*
☐	☒	A description of all covariates tested
☐	☒	A description of any assumptions or corrections, such as tests of normality and adjustment for multiple comparisons
☐	☒	A full description of the statistical parameters including central tendency (e.g. means) or other basic estimates (e.g. regression coefficient) AND variation (e.g. standard deviation) or associated estimates of uncertainty (e.g. confidence intervals)
☐	☒	For null hypothesis testing, the test statistic (e.g. *F*, *t*, *r*) with confidence intervals, effect sizes, degrees of freedom and *P* value noted<br>*Give P values as exact values whenever suitable.*
☒	☐	For Bayesian analysis, information on the choice of priors and Markov chain Monte Carlo settings
☒	☐	For hierarchical and complex designs, identification of the appropriate level for tests and full reporting of outcomes
☒	☐	Estimates of effect sizes (e.g. Cohen's *d*, Pearson's *r*), indicating how they were calculated

Our web collection on statistics for biologists contains articles on many of the points above.

Software and code

Policy information about availability of computer code

Data collection	The intensity of fluorescence per area was calculated by Image Pro plus 6.0 software (Media Cybernetics, MD, USA).
Data analysis	Sample size for each experiment was calculated by analyzing preliminary-experimental data with the PASS 11.0 statistical software (NCSS, UT, USA). Data were collected using Microsoft Excel 16.0.13901.20400 (Microsoft, WA, USA). Data were graphed using Graph Pad Prism 7.0 (GraphPad Software, CA, USA).

For manuscripts utilizing custom algorithms or software that are central to the research but not yet described in published literature, software must be made available to editors and reviewers. We strongly encourage code deposition in a community repository (e.g. GitHub). See the Nature Portfolio guidelines for submitting code & software for further information.

Data

Policy information about availability of data

All manuscripts must include a data availability statement. This statement should provide the following information, where applicable:

- Accession codes, unique identifiers, or web links for publicly available datasets
- A description of any restrictions on data availability
- For clinical datasets or third party data, please ensure that the statement adheres to our policy

All data needed to evaluate the conclusions in the paper are present in the paper and/or the Supplementary Materials. Additional data related to this paper may be requested from the authors. Transcriptome data in this study are available at GSE160914 study in NCBI Gene Expression Omnibus (GEO database). For online accession, please visit: https://www.ncbi.nlm.nih.gov/geo/query/acc.cgi?acc=GSE160914

March 2021

Field-specific reporting

Please select the one below that is the best fit for your research. If you are not sure, read the appropriate sections before making your selection.

☒ Life sciences ☐ Behavioural & social sciences ☐ Ecological, evolutionary & environmental sciences

For a reference copy of the document with all sections, see nature.com/documents/nr-reporting-summary-flat.pdf

Life sciences study design

All studies must disclose on these points even when the disclosure is negative.

Sample size	The sample size was calculated by analyzing our pre-experimental data with the PASS 11.0 statistical software (NCSS, UT, USA).
Data exclusions	Variables that exceed three times the standard deviation were excluded from the analyses.
Replication	Reproductivity is verified using duplicated experiments.
Randomization	For animal experiments, rats with the same ages and weights were grouped .
Blinding	For animal experiments, blinding was introduced for blood pressure measuring operators and the data analyst.

Reporting for specific materials, systems and methods

We require information from authors about some types of materials, experimental systems and methods used in many studies. Here, indicate whether each material, system or method listed is relevant to your study. If you are not sure if a list item applies to your research, read the appropriate section before selecting a response.

Materials & experimental systems

n/a	Involved in the study	
☐	☒	Antibodies
☒	☐	Eukaryotic cell lines
☒	☐	Palaeontology and archaeology
☐	☒	Animals and other organisms
☒	☐	Human research participants
☒	☐	Clinical data
☒	☐	Dual use research of concern

Methods

n/a	Involved in the study	
☒	☐	ChIP-seq
☒	☐	Flow cytometry
☒	☐	MRI-based neuroimaging

Antibodies

Antibodies used	Primary polyclonal chicken anti-MAP2 (1:4000, NB300-213, Novus biological, CO, USA); monoclonal mouse anti-SMI312 (1:3000; #837904, Biolegend, CA, USA); polyclonal rabbit anti-NeuN (1:100; ab177487, abcam, MA, USA) ; rabbit anti-CGRP (1:100; #14959, CST, MA, USA); monoclonal mouse anti-c-Fos (1:100; ab208942, abcam, MA, USA); and rabbit anti-TRPV1 (1:100; ACC-030, alomone labs, Israel) were used. Secondary antibodies used in this study include: Goat anti-chicken IgG/Alexa Flour 488 (bs-0310G-AF488, Bioss Antibodies, China), Alexa Fluor® 488 AffiniPure Goat Anti-Rabbit IgG (H+L) (111-545-003, Jackson Immunoresearch, PA, USA), Cy™3 AffiniPure Goat Anti-Rabbit IgG (H+L) (111-161-003, Jackson Immunoresearch, PA, USA), Goat anti-mouse IgG/Alexa Flour 488 (GB25301, Servicebio, China).
Validation	1. Chicken anti-MAP2 (1:4000, NB300-213, Novus biological, CO, USA): https://www.novusbio.com/products/map2-antibody_nb300-213<br>2. Mouse anti-SMI312 (1:3000; #837904, Biolegend, CA, USA): https://www.biolegend.com/en-us/products/purified-anti-neurofilament-marker-pan-axonal-cocktail-12811<br>3. Rabbit anti-NeuN (1:100; ab177487, abcam, MA, USA): https://www.abcam.cn/neun-antibody-epr12763-neuronal-marker-ab177487.html<br>4. Rabbit anti-CGRP (1:100; #14959, CST, MA, USA): https://www.cellsignal.cn/products/primary-antibodies/cgrp-d5r8f-rabbit-mab/14959?site-search-type=Products&N=4294956287&Ntt=14959&fromPage=plp&_requestid=1095601<br>5. Mouse anti-c-Fos (1:100; ab208942, abcam, MA, USA): https://www.abcam.cn/c-fos-antibody-2h2-ab208942.html<br>6. Rabbit anti-TRPV1 (1:100; ACC-030, alomone labs, Israel): https://www.alomone.com/p/anti-trpv1/ACC-030<br>7. Goat anti-chicken IgG/Alexa Flour 488 (bs-0310G-AF488, Bioss Antibodies, China): http://www.bioss.com.cn/prolook_03.asp?id=AF08169606011692&pro37=4<br>8. Alexa Fluor® 488 AffiniPure Goat Anti-Rabbit IgG (H+L) (111-545-003, Jackson Immunoresearch, PA, USA): https://www.jacksonimmuno.com/catalog/products/111-545-003<br>9. Cy™3 AffiniPure Goat Anti-Rabbit IgG (H+L) (111-161-003, Jackson Immunoresearch, PA, USA): https://www.jacksonimmuno.com/catalog/products/111-161-003

10. Goat anti-mouse IgG/Alexa Flour 488 (GB25301, Servicebio, China): https://www.servicebio.cn/goodsdetail?id=272

Animals and other organisms

Policy information about studies involving animals; ARRIVE guidelines recommended for reporting animal research

Laboratory animals	Spontaneously hypertensive rats (SHR), Wistar-Kyoto rats (WKY) (12 weeks old, weighing about 250 g) and Sprague-Dawley (SD) rats (6 weeks old, weighing about 170 g; or 9 weeks old, weighing about 310 g) were purchased from Vital River Biological Co., Ltd (Beijing, China). All rats were kept in SPF environment, with a temperature of 22±1 degree Celcius and a humidity of 30-60%.
Wild animals	No wild animals were used in this study.
Field-collected samples	The study did not involve samples collected from the field.
Ethics oversight	All animal experiments were approved by the Experimental Animal Care and Use Committee of Nanjing Medical University and performed in accordance with the Guide for the Care and Use of Laboratory Animals (NIH publication No. 85-23, revised 1996).

Note that full information on the approval of the study protocol must also be provided in the manuscript.
